# Targeting FAcilitates Chromatin Transcription complex inhibits pleural mesothelioma and enhances immunotherapy

**DOI:** 10.1186/s13046-023-02889-6

**Published:** 2023-11-16

**Authors:** Anand Singh, Nathanael Pruett, Shivani Dixit, Sudheer K. Gara, Haitao Wang, Roma Pahwa, David S. Schrump, Chuong D. Hoang

**Affiliations:** 1 Thoracic Surgery Branch, National Cancer Institute, National Institutes of Health, Bethesda, MD USA; 2 Urologic Oncology Branch, National Cancer Institute, National Institutes of Health, Bethesda, MD USA

**Keywords:** Mesothelioma, Tumor suppressor, p53, NF-κB, Immunotherapy, Curaxin, CBL0137, FACT complex

## Abstract

**Background:**

Diffuse pleural mesothelioma (DPM) is an aggressive therapy-resistant cancer with unique molecular features. Numerous agents have been tested, but clinically effective ones remain elusive. Herein, we propose to use a small molecule CBL0137 (curaxin) that simultaneously suppresses nuclear factor-κB (NF-κB) and activates tumor suppressor p53 via targeting FAcilitates Chromatin Transcription (FACT) complex, a histone chaperone critical for DNA repair.

**Methods:**

We used DPM cell lines, murine models (xeno- and allo-grafts), plus DPM patient samples to characterize anti-tumor effects of CBL0137 and to delineate specific molecular mechanisms.

**Results:**

We verified that CBL0137 induced cell cycle arrest and apoptosis. We also discovered that DPM is a FACT-dependent cancer with overexpression of both subunits structure-specific recognition protein 1 (SSRP1), a poor prognosis indicator, and suppressor of Ty 16 (SUPT16H). We defined several novel uses of CBL0137 in DPM therapy. In combination with cisplatin, CBL0137 exhibited additive anti-tumor activity compared to monotherapy. Similarly, CBL0137 (systemic) could be combined with other novel agents like microRNA-215 (intrapleural) as a more effective regimen. Importantly, we established that CBL0137 induces immunogenic cell death that contributes to activating immune response pathways in DPM. Therefore, when CBL0137 is combined with dual immune checkpoint inhibitors DPM tumor growth is significantly suppressed.

**Conclusions:**

We identified an unrecognized molecular vulnerability of DPM based on FACT dependency. CBL0137 alone and in several combinations with different therapeutics showed promising efficacy, including that of improved anti-tumor immunity. Overall, these preclinical findings suggest that CBL0137 could be ideally suited for use in DPM clinical trials.

**Supplementary Information:**

The online version contains supplementary material available at 10.1186/s13046-023-02889-6.

## Background

Diffuse pleural mesothelioma [1] (DPM) is a recalcitrant surface membrane neoplasm that, contrary to historical predictions, continues to remain prevalent worldwide with increasing incidence [2]. Conventional drug therapy in DPM has been disappointing with a dismal median survival time of 12–18 months [3].

Recently, the CheckMate 743 trial (NCT02899299) established nivolumab, a fully human anti-programmed death-1 (PD-1) antibody, plus ipilimumab, a fully human anti-cytotoxic T-lymphocyte antigen-4 (CTLA-4) antibody, as first-line therapy in unresectable DPM [4]. However, only ~ 40% of patients exhibited a partial response from this dual immune checkpoint inhibitor (ICI) therapy, and 30% of the patients developed grade 3–4 treatment-related adverse events. In subgroup analysis, those with sarcomatoid histology, the least common, benefited more than those with epithelioid histology, the most common variety. Mature 3-year outcomes from this trial indicate the need for further improvements. With the combination ICI, the median overall survival (OS) was 18.1 months, the 3-year OS rate was 23%, and objective response rates of 40% [5]. Additionally, only 8% remained on ICI for the planned 2-year treatment course, while 44% switched to chemotherapy. Hence, with only two FDA-approved anti-DPM drug regimens [4, 6], there is a compelling, ongoing need for more effective treatments. In fact, a recent comparative effectiveness study analyzing the statistical robustness of all the phase III trials in DPM encompassing 1501 patients was not able to conclude any real survival benefits for any drug regimens [7].

Contemporary genetic profiling has largely reaffirmed known characteristics of DPM pathobiology, which entail a low protein-altering mutation rate with a predominance of tumor-suppressor gene losses, yet a lack of oncogenic driver mutations [8]. Unique and pervasive molecular hallmarks of DPM include predominantly wild-type *TP53* (8% mutated entirely in non-epithelioid tumors [8]) and dependency on chronic inflammation effects associated with asbestos exposure, ultimately mediated by nuclear factor-κB (NF-κB) signaling [9, 10].

Various therapeutic strategies have attempted to leverage these DPM molecular vulnerabilities. Inhibitors of Murine Double Minute 2 (MDM2), the main negative regulator of tumor suppressor p53, were tested in phase I settings. Although there is supporting preclinical data for these drugs in DPM [11], such results have never reached to clinical settings for DPM patients [12]. Anti-inflammatory approaches in MPM specifically targeting NF-κB were disappointing. Namely, the proteosomal inhibitor bortezomib that antagonizes NF-κB [13], ultimately failed to meet endpoints in phase II trials where its therapeutic efficacy on DPM was investigated alone [14] or in combination with cisplatin [15].

In this context, we posit there are more efficient choices of drug therapy for DPM. In fact, there is a second-generation curaxin or CBL0137 [1,1′-(9-(2-(isopropylamino)ethyl)-9H-carbazole-3,6-diyl)bis(ethan-1-one)] that intercalates DNA causing torsional stress. This mechanism traps the histone chaperone FAcilitates Chromatin Transcription (FACT) complex, leading to activation of p53 by phosphorylation at serine 392 (Ser^392^) by binding casein kinase 2 and simultaneous inhibition of NF-κB-dependent transcription [16]. FACT is a heterodimer of two subunits structure-specific recognition protein 1 (SSRP1) and suppressor of Ty 16 (SUPT16H). Because CBL0137 has entered human trials [17], our objective was to verify its efficacy alone or in novel combinations against DPM using several preclinical models.

## Methods

### Reagents

The CBL0137 hydrochloric acid (curaxin) and cisplatin were purchased from Selleckchem. Monoclonal anti-mouse PD-1 (CD279) antibody (#BE0146), monoclonal rat IgG2a isotype control, anti-trinitrophenol antibody (#BE0089), monoclonal anti-mouse CTLA-4 (CD152) antibody (#BE0131), and polyclonal Syrian hamster IgG, isotype control antibody (#BE0087) were purchased from BioXcell, USA.

### Tissues and cell culture

Human specimen collection followed our institutional review board (IRB)-approved protocols. All surgical specimens were de-identified and stored at − 80 °C. We selected 50 DPM tumors of all three histologies and 31 non-patient-matched, benign pleuras based on the amounts of usable tissue available. Human DPM cell lines, H2052, H2373, H2596, H2452, and MSTO-211H were obtained from ATCC. Low passage, patient-derived DPM cell lines (Supplemental Table 1), #24 (MB24), #34 (MB34), #52 (MB52), and #8 T (MB8T) were obtained from Mesobank UK, an international bioresource [18, 19]. Murine DPM cell lines (AB1, AB22, and AB12) [20] were purchased from Sigma. All cell lines were cultured as monolayers at 37 °C and 5% CO2 in RPMI 1640 supplemented with 10% fetal bovine serum (FBS) and 1% penicillin/streptomycin solution. A patient-derived normal pleural mesothelial cell line NP1 was cultured and maintained as described previously [21]. All cell culture media, media supplements, and FBS were purchased from Thermo Fisher Scientific. All datasets used and/or analyzed during the current study are available from the corresponding author on reasonable request.

### Quantitative Real-time Polymerase Chain Reaction (qRT-PCR)

Total RNA was isolated cell lines and from tissue specimens using TRIzol™. Quantification of RNA was done using a NanoDrop 2000 spectrophotometer. For mRNA analysis, total RNA was reverse-transcribed using the High-Capacity cDNA Reverse Transcription Kit. Expression of mRNA was determined by TaqMan® analysis on a QuantStudio 6 Flex PCR system [22]. The expression of a gene was normalized to β-actin. qRT-PCR primers used for the analysis of gene expression were available from Applied Biosystems (Supplemental Table 4). All qRT-PCR reactions were performed in triplicate. All reagents and equipment are from Thermo Fisher Scientific.

### Cell viability

DPM cells were seeded into 96-well plates at a density of 3.0 × 10^3^ cells/well. Under several experimental conditions, cell viability was measured using the CellTiter 96® AQueous One Solution Cell Proliferation Assay (Promega). Studies were performed in three independent cell preparations.

### Clonogenicity

Colony foci formation (2D) was assessed in vitro. Briefly, 1.0 to 1.5 × 10^3^ DPM cells/ well were seeded in 12-well plates. After 24 h, DPM cells were treated with CBL0137 with different concentrations. Culture media was replenished every third day. After 12 to 14 days, colony foci were stained with crystal violet (0.5%) and imaged using the Bio-Rad gel imager.

### Western blot analysis

DPM cells were lysed in radio-immunoprecipitation buffer (RIPA) and their protein concentration was determined using the bicinchoninic acid protein assay kit (Thermo Fisher Scientific). Protein lysates were fractionated on 4 to 15% polyacrylamide gels and transferred onto nitrocellulose (Bio-Rad). These primary antibodies were used: β-actin (#8457), p21 (#2947), NF-κB p65 (#8242), phospho-p53 (Ser^392^) (#9281), cleaved caspase-3 (#9661), PARP (#9542) all at 1:1000 dilution (Cell Signaling Technology); additionally, p53 (sc-126) at 1:200 dilution (Santa Cruz Biotechnology). Secondary antibodies anti-rabbit and anti-mouse conjugated with horseradish peroxidase were used for the detection of protein signal (Abcam). All protein experiments were performed in triplicate.

### Cell cycle analysis

Flow cytometric analysis of DNA content in fixed DPM cells was performed using the FxCycle™ PI/RNase Staining Solution according to manufacture instructions (Thermo Fisher Scientific). Briefly, into each well of a 6-well plate, 2.0 × 10^5^ DPM cells were seeded and after 24 h treated with CBL0137 (300 nM) or control (DMSO). Following 48 h of treatment, data were acquired and analyzed on a BD FACSCalibur™ platform according to manufacturer’s recommendations (BD Biosciences). Experiments were performed in triplicate.

### Apoptosis analysis

DPM cells at a density of 2.0 × 10^5^ cells/ well were treated with control (DMSO) or CBL0137 (300 nM) in 6-well plates for 72 h. Both adherent and floating cells were collected. Apoptotic or dead cells were detected by fluorescence-activated cell sorting (FACS) analysis using the Annexin V-FITC Apoptosis detection Kit (Abcam) according to manufacturer’s instructions.

### Cell transfection

DPM cells were transfected with miR-215-5p mimic or mirVana™ miRNA mimic Negative Control #1 were transfected into cell lines at a final concentration of 20 nM using Lipofectamine® RNAiMAX according to manufacturer’s instructions (Thermo Fisher Scientific).

### NF-κB- and p53-luciferase reporter assay

To verify the effect of CBL0137 on NF-κB and p53 activity, NF-κB luciferase reporter (pGL4.32[luc2P/NF-κB-RE/Hygro]) and p53-luciferase reporter (pGL4.38[luc2P/p53 RE/Hygro]) constructs driving the downstream luciferase reporter gene expression, were used (Promega). In brief, DPM cells were seeded into 6-well plates at a density of 2.0 × 10^5^ cells/well. Next day, these cells were co-transfected with NF-κB/p53-luciferase reporter construct and control reporter vector (pRL-TK) using Lipofectamine 2000 with Opti-MEM (Thermo Fisher Scientific). After 24 h of transfection, cells were treated with CBL0137 or control (DMSO) at indicated experimental conditions. Following 24 h of treatment, cells were harvested, and the relative luciferase activity (firefly/renilla) was measured by Dual-Luciferase Reporter Assay kit (Promega).

### Immunogenic cell death assays

Cells at a density of 2.0 × 10^5^ cells/ well were treated with control (DMSO) or CBL0137 (300 nM) in 6-well plates for 72 h and supernatants were collected. Cells were counted to quantify secreted ATP and HMGB1. ATP concentration was determined by using ATP Determination Kit (Thermo Fisher Scientific, A22066) and HMGB1 by HMGB1 ELISA kit (IBL international, Tecan, ST51011). For detection of cell surface expression of CALR, human DPM cells were stained with anti-CALR Alexa Fluor® 488-conjugated antibody (R&D systems, IC38981G) or mouse IgG2B Alexa Fluor® 488-conjugated isotype control (R&D systems, IC0041G), while mice DPM cells were stained with anti-CALR (D3E6) XP® Rabbit Alexa Fluor® 488-conjugated antibody (Cell signaling technology, #62,304) or Rabbit (DA1E) mAb IgG XP®, Alexa Fluor® 488 conjugated, isotype control (Cell signaling technology, #2975) according to manufacturer’s protocols, and analyzed by flow cytometry.

### PD-L1 Flow cytometry analysis

PD-L1 cell surface expression was measured on normal mesothelial cells and DPM cells by staining with the Alexa Fluor® 488 Anti-PD-L1 antibody (Abcam, ab209959) or Alexa Fluor® 488 Rabbit IgG, monoclonal [EPR25A]- Isotype control (Abcam, ab199091) according to manufacturer’s instructions. Data were acquired and analyzed on FACSCalibur™ flow cytometer (BD Biosciences).

### RNA-seq analyses

Sequencing data quality was assessed using FastQC version 0.11.5. Raw reads with low quality were removed using Trim Galore version 0.4.4 prior to data analysis. The trimmed reads were aligned to the human reference genome (GRCh38) using STAR version 020201. The mapped read counts were extracted using the feature count tool from the Subread package version 1.5.3. Read count data contained 53,717 quantified transcripts (coding and non-coding). Genes with zero read count across all samples were filtered out, leaving 50,423 genes (coding and non-coding) for downstream analysis. The read count data were normalized using DESeq2 [23].

To identify differentially expressed genes (DEG) between CBL0137 treated and control DPM cell lines, we applied a filter of |log_2_ fold change|> 1 and calculated p-values using moderated t-statistics implemented in the Limma package. Gene-set enrichment analysis (GSEA) was performed using the Bioconductor R package clusterProfiler [24]. Gene sets from the Cancer hallmark, Kyoto Encyclopedia of Genes and Genomes (KEGG), and Gene Ontology (GO) were collected from Molecular Signature Database (MSigDB 7.0). Functional enrichment analysis was performed on upregulated and downregulated genes in DPM cell lines.

### Mice xenografts and allografts

Animal experiments were approved by our Animal Care and Use Committee in accordance with NIH Guidelines. For subcutaneous xenografts, H2373 or MB52 cells (3.0 × 10^6^) were injected into the right flank of NOD.Cg-PrkdcscidIl2rgtm1Wjl/SzJ (NSG) male mice (6–10 weeks old) [25]. When tumor volume reached an average of 100–150 mm^3^, mice were treated under several experimental conditions. CBL0137 was dissolved in Captisol (CyDex Pharmaceuticals) for in vivo experiments. H2373 or MB52 cells (1.0 × 10^6^) stably expressing the luciferase reporter vector pGL4.51[luc2/CMV/Neo] (Promega) were implanted into the pleural space of NSG mice (6–8 weeks old). After one week, mice were randomized and divided into different treatment conditions as indicated. For miR-215-5p and CBL0137 combinations, our peptide-based SFH nanocomposite delivered miR-215-5p or control miRNA mimic intrapleurally as described previously [26], followed by CBL0137 systemic administration. Syngeneic AB1-cells (1.0 × 10^6^) were injected subcutaneously into the right flank of 6–10 weeks old BALB/cJ male mice (The Jackson Laboratory). Once tumor volume reached an average of 100–120 mm^3^, mice were randomized and divided into the several experimental groups as indicated in the results. As indicated, tumor growth was monitored three times per week by measuring the length (L), width (W), and height (H) of each tumor using calipers. Volumes were calculated using the equation (LxWxH)^1/3^ (mm^3^).

### Immunohistochemistry

Tumor tissues were fixed in 10% neutral buffered formalin for 48 h and transferred to 70% ethanol before paraffin processing. Tissues sections were cut in 5 µm-thick sections, deparaffinized, serially rehydrated in ethanol solutions and followed by antigen retrieval with heated citrate buffer (Vector Laboratories, H3300) according to manufacturer’s instructions. Primary antibodies consisted of the following: anti-cleaved caspase-3, 1:200 (Cell Signaling Technology, 9661), anti-PD-L1, 1:200 (Cell Signaling Technology, 64,988), anti-CD3, 1:200 (Abcam, ab16669), and anti-CD8, 1:200 (Abcam, ab209775). Pathologic slides were developed with the horseradish peroxidase/ diaminobenzidine (ABC) Detection immunohistochemistry kit (Abcam, ab64264), per the manufacturer’s instructions. Hematoxylin was the Counterstain (Vector Laboratories, H-3404). Tissue sections were mounted in VectaMount AQ Aqueous Mounting Medium (Vector Laboratories, H-5501), and images were acquired on an automated digital slide scanner (Aperio ScanScope XT, Leica Biosystems).

### Statistics

Means and standard error of the mean (SEM) or standard deviation (SD) were calculated from numerical data. Changes (fold or percentage) indicate the difference between experimental and control groups. As indicated, values in bar graphs are presented as the mean ± SEM or ± SD. Two-tailed, unpaired Student’s *t* test assessed significance between two conditions. Nonparametric Mann–Whitney test compared the differences in genes (mRNA) expression between normal pleura and DPM tumor specimens. One-way analysis of variance (ANOVA) was used to determine statistical significance between the means of multiple groups. Kaplan–Meier and log-rank test were applied for survival analysis. A p-value < 0.05 was considered statistically significant. GraphPad Prism v9.3.1 software was used for statistical calculations.

## Results

### DPM is associated with FACT Subunits SSRP1 and SUPT16H overexpression

**Fig. 1:**
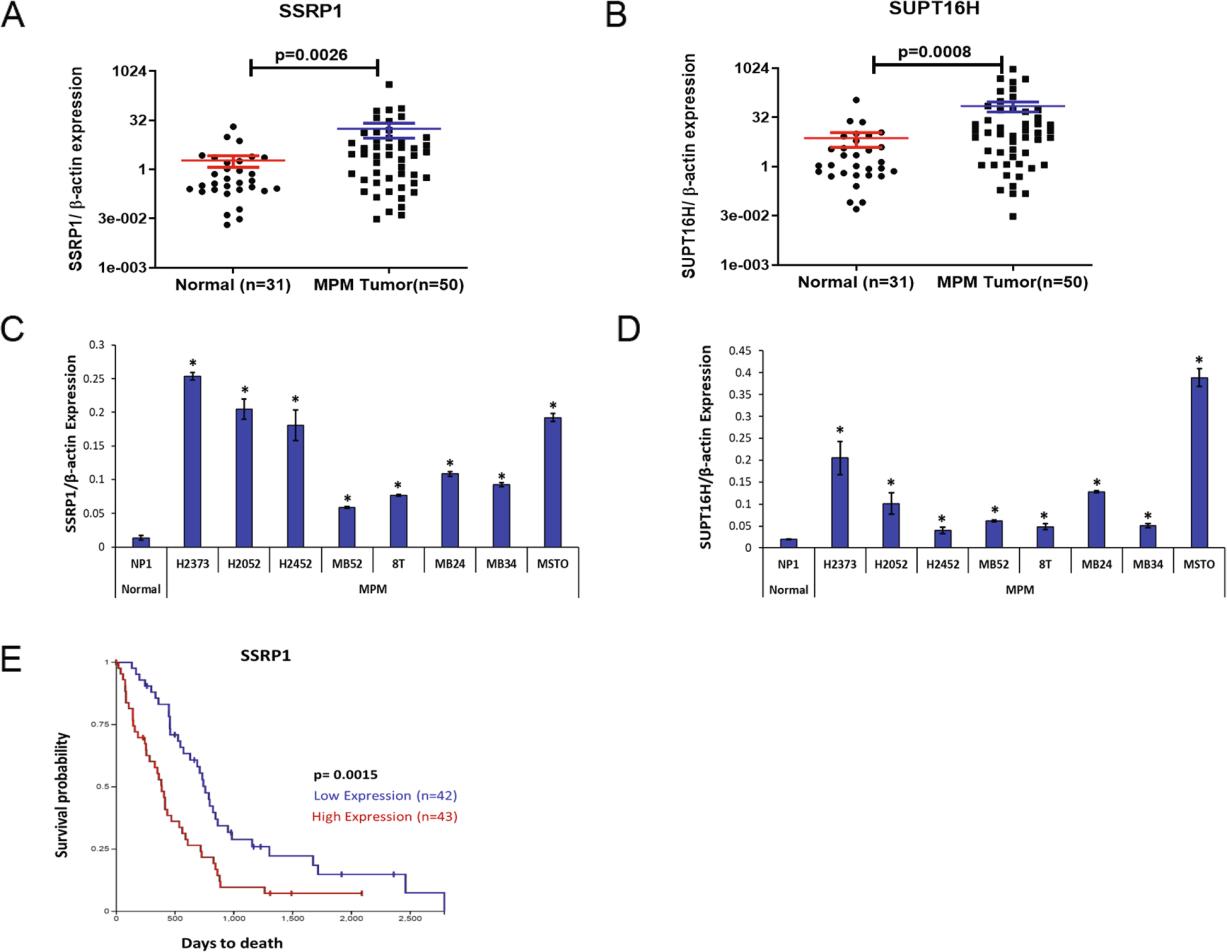
Expression of FACT subunits in mesothelioma and prognostic implications. **A**-**B** SSRP1and SUPT16H transcript abundance in DPM was quantified by qRT-PCR. The tumors tested were largely represented by epithelioid histology. **C**-**D** SSRP1and SUPT16H transcript abundance in DPM cell lines (all histologies are represented) versus the normal mesothelial cell line NP1. **E** Kaplan–Meier analysis of TCGA-meso data showing overall survival of DPM patients based on dichotomized expression of SSRP1 (high or low relative to its median expression). Dot plots are expressed as mean ± SEM; bar graphs represent the mean ± SD of triplicates; p values in tissue samples were calculated by the Mann–Whitney test and in cell lines by the two-tailed Student’s *t* test. *p < 0.05 is considered significant

To evaluate any potential relevance of CBL0137 for DPM therapy, we first ascertained expression levels of the FACT complex subunits in tissues and cells. We examined the SSRP1 and SUPT16H transcripts in available surgical MPM specimens (*n* = 50 randomly selected; 46 epithelioid, three biphasic, and one sarcomatoid) compared with unmatched normal pleuras (*n* = 31) using quantitative real-time PCR (Supplementary Table 2). The transcript abundance of SSRP1 and SUPT16H was each significantly upregulated in DPM tissues by 9.23- (*p* = 0.0026) and 19.16- (*p* = 0.0008) fold, respectively, compared to normal pleura (Fig. 1A and B). This overexpression pattern was mirrored in a panel of DPM cell lines (Fig. 1C and D).

Furthermore, we interrogated The Cancer Genome Atlas (TCGA)-meso data (https://portal.gdc.cancer.gov/; *n* = 85 specimens; Supplementary Table 2) for any associations of FACT complex expression with outcomes. High expression of SSRP1, the subunit most commonly cancer-related and specifically the target of drugs [17], is associated with poor OS (*p* < 0.01) by Kaplan–Meier analysis (Fig. 1E). Because TCGA does not include normal samples as a reference, high versus low expression of SSRP1 in this analysis is calculated relative to its median expression among the entire DPM cohort. Taken together, these data form a basis for FACT inhibition as a novel DPM therapy.

### CBL0137 inhibits NF-κB and activates p53 to induce apoptosis and cell cycle arrest in DPM cells

**Fig. 2:**
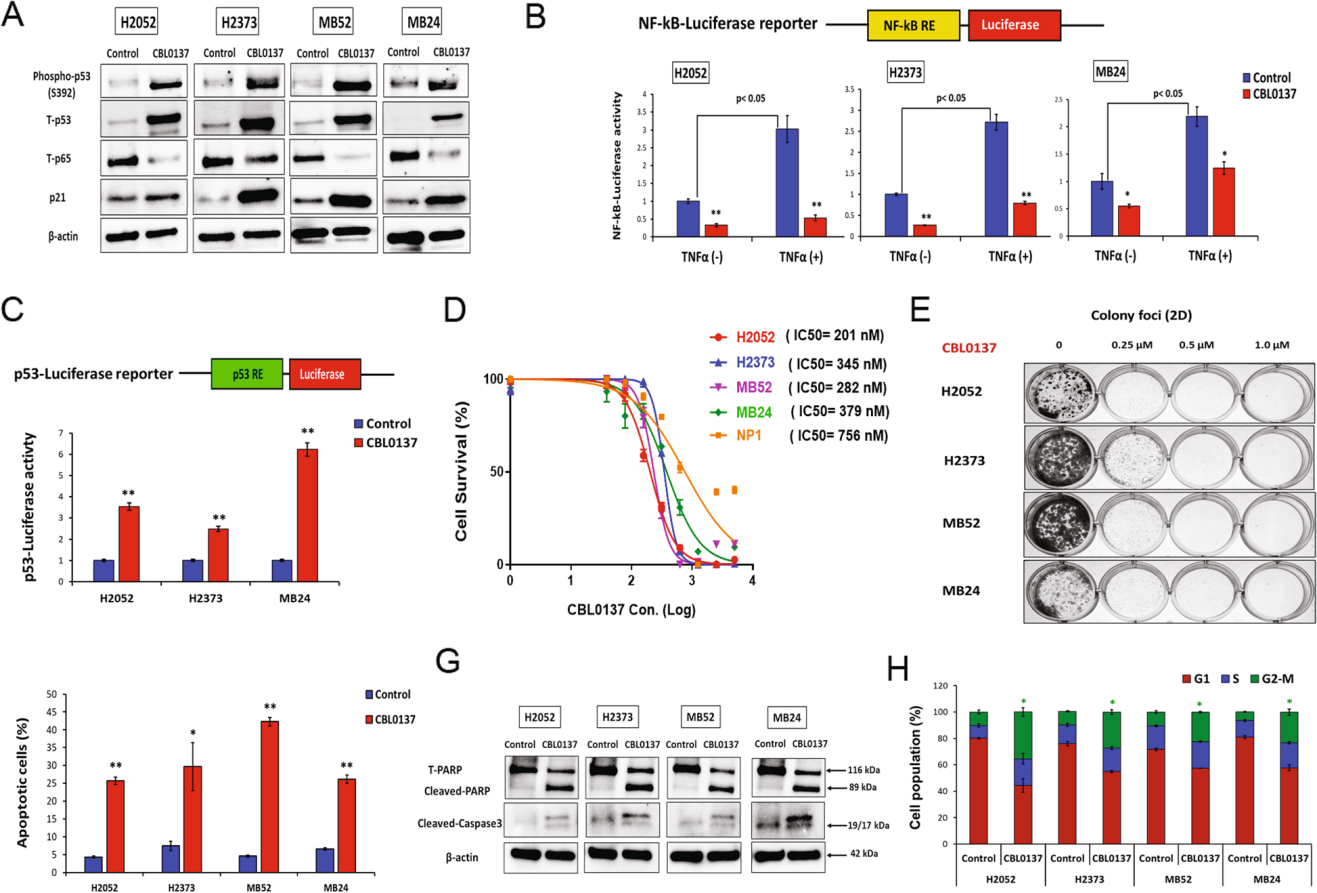
CBL0137 mechanism of action (NF-κB inhibition and p53 activation) and effects on mesothelioma cells. **A** DPM cells were treated with CBL0137 (300 nM) for 48 h. Western blots revealed differences in the levels of total p53, phosphorylated-p53 (Ser^392^), total NF-κB (p65), and p21 for treated versus untreated (control) cells. **B** Luciferase activity was measured in DPM cells transfected with NF-κB-luciferase reporter construct followed by 24 h of CBL0137 (300 nM) treatment with or without TNFα (100 ng/ml) stimulation. **C** Luciferase activity was assessed in DPM cells transfected with p53-luciferase reporter vector followed by 24 h of CBL0137 (300 nM) treatment. **D** Cell survival was measured in DPM cells treated with increasing concentrations of CBL0137 for 72 h. Data are expressed as means ± SD of quadruplicates and are representative of three independent experiments. **E** Colony foci (2D) formation in DPM cells following CBL0137 treatment. Data are representative of three independent experiments. **F** Annexin-V staining using flow cytometry after 72 h of CBL0137 treatment. **G** Western blot analysis of cleaved PARP and caspase-3 levels (apoptosis markers) in DPM cells treated with CBL0137 (300 nM) or control at 72 h. Data are representative of two independent experiments. **H** Cell cycle distribution was determined by flow cytometry in DPM cells treated with CBL0137 (300 nM) or control at 48 h. Bar graphs show the cell population percentage of DPM cells after treatment. Where applicable, data are presented as means ± SEM, *n* = 3. *p < 0.05; **p < 0.01; IC50, half maximal inhibitory concentration; PARP, poly (ADP-ribose) polymerase

The FACT signature in DPM tissues suggested that therapeutic targeting of this complex would result in tumor-specific inhibitory effects. CBL0137 by intercalating DNA, traps FACT complex and initiates multiple effects on factors like NF-κB and p53, but does not specifically target its subunits [16]. Consequently, by Western blot analysis of DPM cell lines, CBL0137 treatment inhibited NF-κB (p65) levels while it increased total p53 as well as phosphorylated-p53 (Ser^392^) levels compared to control (Fig. 2A). Evidence of specific targeting of NF-κB (inhibition) and p53 (activation) by CBL0137 was verified using luciferase reporter assays (Fig. 2B and C). Dose–response characteristics of CBL0137 revealed high potency in killing epithelioid and sarcomatoid DPM cells, only requiring very low half-maximal inhibitory concentrations (IC_50_) in the range of 200 to 380 nM (Fig. 2D). This killing effect was relatively more specific for DPM since the IC_50_ for the NP1 cells (control) was nearly double at 756 nM. CBL0137 treatment significantly suppressed the colony-forming ability (clonogenicity) of DPM cells in a dose-dependent manner compared to control (Fig. 2E).

The molecular mechanism of CBL0137 implicates apoptosis and cell cycle effects in susceptible DPM cells. The percentage of apoptotic cells, quantitated by annexin-V flow cytometry, increased in treated DPM cells compared to control (*p* < 0.05) (Fig. 2 and Supplementary Fig. 1A). Significantly increased caspase-3/7 activity (Supplementary Fig. 1B) and Western blots showing more abundant cleaved poly (ADP-ribose) polymerase (PARP) and caspase-3 (Fig. 2G) (pro-apoptotic markers) in treatment versus controls confirmed that CBL0137 induced apoptosis in DPM. Furthermore, cell cycle arrest in G2-M phase was more prevalent in treatment compared to control (Fig. 2J and Supplementary Fig. 2). Collectively, these results indicate that CBL0137 greatly diminished malignant features in DPM cells.

### CBL0137 suppresses DPM in vivo

**Fig. 3:**
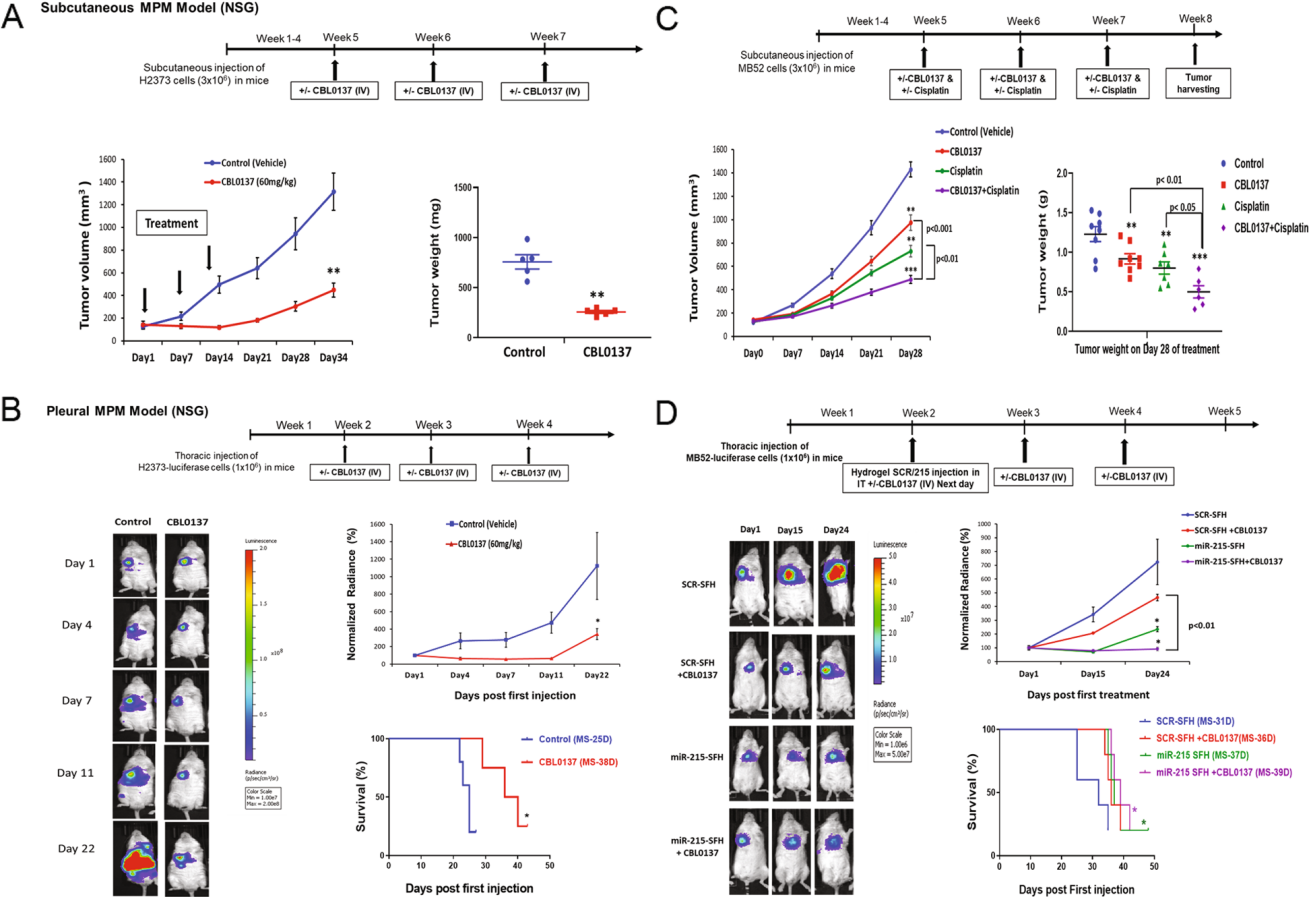
In vivo effects of CBL0137 as mesothelioma monotherapy and in novel therapeutic combinations. **A** H2373 cells were implanted in the flanks of NSG mice to establish subcutaneous xenografts. Once tumor volume reached an average of 130 mm^3^, mice were randomized and divided in two groups. Mice were injected with CBL0137 or delivery vehicle intravenously once a week until 3 weeks (*n* = 5 in each group). Graph depicts the changes in tumor volume and weight over 5 weeks after first injection of drug. **B** The schematic timeline of monitoring tumor growth in mice bearing intrathoracic H2373-luciferase tumor cells followed by CBL0137 or vehicle control intravenous injection once a week until 3 weeks. Lower left: live imaging shows tumor luminescence in mice treated with CBL0137 or control at the indicated time points (*n* = 5 in each group). Tumor growth curve of mice harboring intrathoracic H2373-luciferase cells in CBL0137 and control treatment groups based on luminescence intensity. OS depicted by Kaplan–Meier curve of mice treated with CBL0137 or control with a log rank (Mantel-Cox) test assessing for significance. **C** The schematic timeline for combination treatment of CBL0137 with cisplatin in DPM xenografts. MB52 cells were implanted in the flanks of NSG mice. Once tumor volume reached an average of 120 mm^3^, mice were randomized and divided in four groups. Mice were treated with drug vehicle (*n* = 8), CBL0137 (20 mg/kg per day via oral gavage, Days 1 and 2 of a week, *n* = 8), cisplatin (3 mg/kg per day; intraperitoneal, once a week, *n* = 7), or a combination of CBL0137 and cisplatin (*n* = 6), at indicated time points. Graphs depicts the changes in tumor volume and weight over 4 weeks after first injection. **D** The schematic timeline of monitoring tumor growth in mice harboring intrathoracic MB52-luciferase tumor cells followed by control miRNA (SCR-SFH) or miR-215-5p mimic (miR-215-SFH) injection once into the pleural cavity of NSG mice alone or in combination with CBL0137 (IV), given in multiple doses. Lower left: live imaging shows tumor luminescence in mice treated with control (SCR-SFH) or miR-215-SFH alone or in combination with CBL0137 at the indicated time points (*n* = 5 in each group). Tumor growth curve of mice harboring intrathoracic MB52-Luciferase cells in different treatment groups based on luminescence intensity. Overall survival depicted by Kaplan–Meier curve of mice treated with SCR-SFH or miR-215-SFH alone or in combination with CBL0137 with a log rank (Mantel-Cox) test assessing for significance. Data are presented as means ± SEM. *p < 0.05; **p < 0.01; ***p < 0.001; all p calculated with the two-tailed Student’s t test or Mann–Whitney test. NSG, NOD.Cg-PrkdcscidIl2rgtm1Wjl/SzJ mice (immunodeficient); IV, intravenously; IT, intrathoracic; SFH, surface-fill hydrogel; SCR-SFH, control miRNA; MS, median survival; D, days

Next, the anti-tumor efficacy of CBL0137 was assessed in DPM tumor models, according to our previous protocols [25]. Subcutaneous DPM human xenografts were established in NOD.*Cg*-*Prkdc*^*scid*^*Il2rg*^*tm1Wjl*^/SzJ (NSG) mice using H2373 cells. CBL0137 or drug vehicle (control) was delivered intravenously (IV) at three time points. CBL0137 treatment significantly suppressed the growth of DPM tumors (Fig. 3A and Supplementary Fig. 3) compared to control group (*p* < 0.01). The efficacy of CBL0137 was also assessed in intrapleural orthotopic xenografts harboring DPM cells stably expressing a luciferase gene to estimate tumor burden by monitoring luminescence. Again, CBL0137 treatment significantly inhibited tumor growth compared with control group and markedly improved OS (median 38 days) compared to control (median 25 days) (all *p* < 0.05) (Fig. 3B). All these data indicate that CBL0137 is efficacious and beneficial as therapy against DPM in preclinical mouse models.

### CBL0137 enhances the efficacy of cisplatin and microRNA-215 as DPM therapy

In exploring the extended clinical utility of CBL0137, we assessed therapeutic efficacy in rational agent combinations based on similar cell killing phenotypes. Like CBL0137, cisplatin induces apoptosis in DPM and is the backbone drug [6] in conventional regimens. Accordingly, we combined CBL0137 with cisplatin and noticed more efficient DPM cell killing at low doses in cell viability assay compared to control or monotherapy (Supplementary Fig. 4A). This additive effect is consistent with prior observations in other tumor models such as medulloblastoma [27]. Additionally, a novel combination of CBL0137 with microRNA (miRNA or miR)-215-5p was tested since this small RNA induces a positive MDM2-p53 signaling loop sustaining p53 activity in DPM [25]. MB52 (epithelioid) cells transfected with miR-215-5p or control miRNA mimic were exposed to increasing concentrations of CBL0137 in a cell viability assay. Cells with miR-215-5p re-expression showed a significantly lower drug IC_50_ level compared to control (Supplementary Fig. 4B), indicating an additive DPM cell killing effect.

The additive effects of these therapeutic combinations were also assessed in vivo using NSG mice. At different time points, subcutaneous xenograft (MB52 cells) groups were treated with drug vehicle (control), CBL0137, cisplatin, or a combination of CBL0137 and cisplatin. Monotherapies significantly suppressed tumor growth compared to control (*p* < 0.01), whereas the combination treatment showed the most pronounced tumor suppression over 4 weeks of observation (*p* < 0.001) (Fig. 3C and Supplementary Fig. 5).

Next, intrapleural orthotopic xenografts were treated with CBL0137 and miRNA. This novel regimen consisted of systemic drug and locally delivered short RNA carried by a new polymer surface-fill hydrogel (SFH) eluting miRNA nanoparticles [26]. The SFH complexed with control miRNA (SCR-SFH) or miR-215-5p mimic (miR-215-SFH) was injected once into the pleural cavity of NSG mice alone or in combination with CBL0137, given in multiple doses (Fig. 3D). CBL0137 and miR-215-SFH monotherapies significantly suppressed DPM compared to control (*p* < 0.05). However, the combination treatment showed more potent tumor suppression (*p *< 0.05), which was associated with the best OS (median 39 days) by Kaplan–Meier analysis. With miR-215-SFH or CBL0137 monotherapy, the median OS was 37 and 36 days, respectively, compared to control (median 31 days). These data illustrate the potential of multiple in vivo combinations with CBL0137 to produce additive cytotoxic effects during DPM therapy.

### CBL0137 targets additional gene pathways

**Fig. 4:**
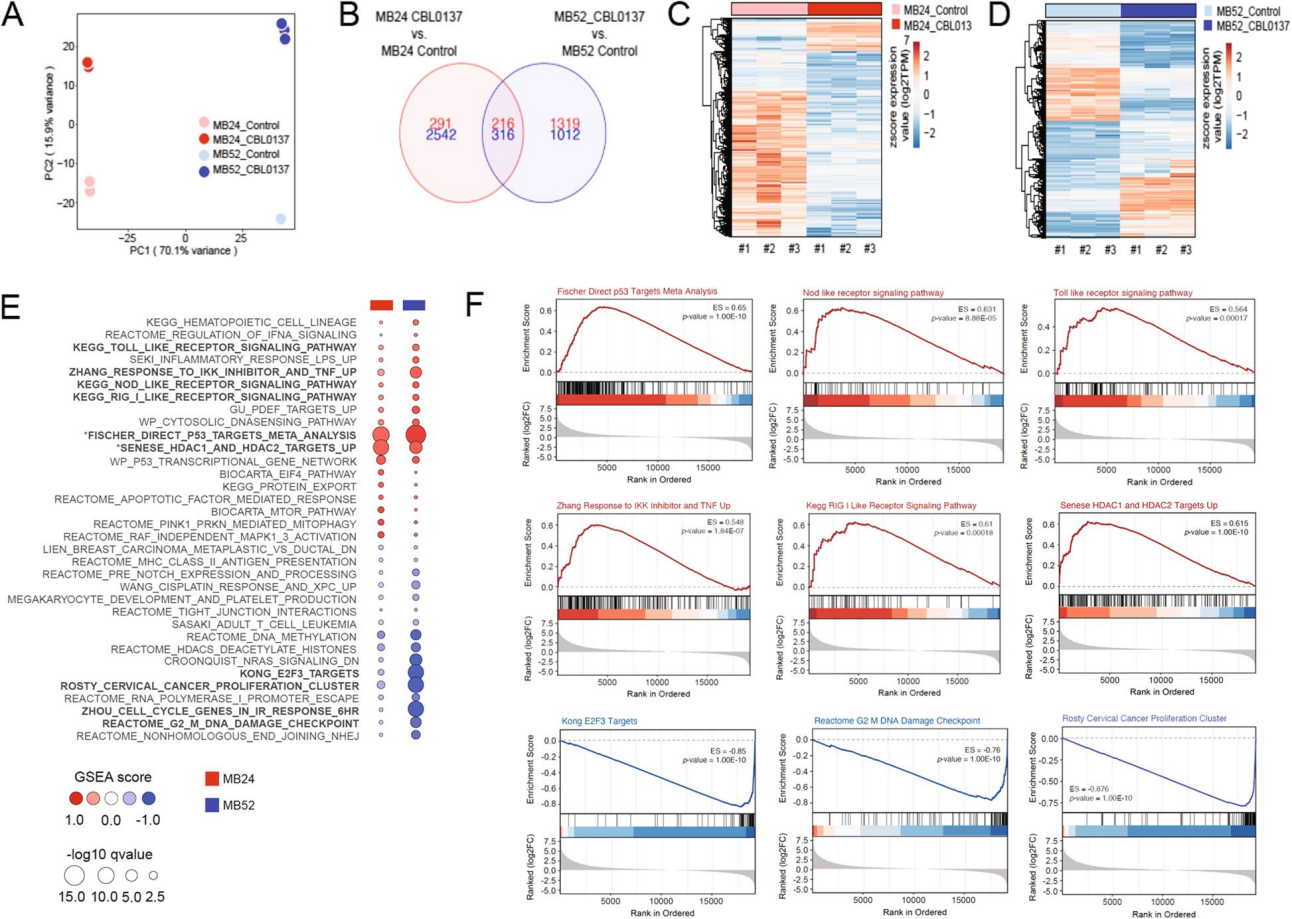
Genome-wide effects of CBL0137 by RNA sequencing of mesothelioma cells. **A** Principal component analysis of mRNA-seq data from MB24 and MB52 cells following 48 h of CBL0137 or control treatment. **B** Venn diagram showing the number of DEG in MB24 and MB52 following treatment with CBL0137 compared to control. Of the 532 DEG common in both cell lines, 316 genes were downregulated, and 216 genes were upregulated. **C**& **D** The heatmap represents the DEG in MB24, and MB52 cells with CBL0137 treatment compared to control. **E** The top ranked (positively and negatively) enriched gene sets were identified using GSEA in response to CBL0137. GSEA was performed with common DEG in MB24 and MB52 cells after CBL0137 treatment using the Cancer hallmark, Kyoto Encyclopedia of Genes and Genomes (KEGG), and Gene Ontology (GO) gene sets from MSigDB database. **F** Representative GSEA plots showing positive enrichment of the Fischer direct p53 targets meta-analysis, Senese HDAC1 and HDAC2 targets up, Zhang response to IKK inhibitor and TNF up, KEGG toll like receptor signaling pathway, KEGG nod like receptor signaling pathway, and KEEG RIG I like receptor signaling. Representative GSEA plots showing negative enrichment for the gene sets involved in cell cycle progression and proliferation including Kong E2F3 Targets, reactome G2 M DNA damage checkpoint, and Rosty cervical cancer proliferation cluster pathway. *GSEA* Gene set enrichment analysis, *ES* Enrichment score

Aside from the core targets of NF-κB and p53, CBL0137 exerts it anti-cancer effects via diverse mechanisms and alternate gene pathways, many of which remain to be fully elucidated [17]. We used next generation RNA-sequencing (RNA-seq) and GSEA to develop DPM-specific exploratory data for leads on other CBL0137 molecular targets of potential relevance. Both epithelioid (MB52 cells) and sarcomatoid (MB24 cells) histologies were treated with CBL0137 and profiled by RNA-seq to derive DEG. Principal component analysis highlighted the dissimilarity of gene expression profiles defining MB24 apart from MB52 cells at baseline and after CBL0137 treatment, which induced significant alterations in global gene profiles compared to their respective control groups (Fig. 4A).

Only 532 DEG (*p* < 0.05; log_2_ fold change > 1) were common in both cell lines, with 316 genes downregulated while 216 genes were upregulated (Fig. 4B and Supplementary Table 3). The heatmap displays DEG in MB24 versus MB52 cells (Fig. 4C and D; Supplementary Fig. 6). These genes were ranked by p-value and fold change between CBL0137 versus control (vehicle) treated cells (Supplementary Table 3). Next, GSEA was performed to identify biological pathways following CBL0137 treatment. We observed multiple gene sets that were significantly enriched, including those associated with p53 activation, NF-κB inhibition, cellular proliferation, and innate immune response (Fig. 4E and F).

### CBL0137 induces immunogenic cell death in DPM

**Fig. 5:**
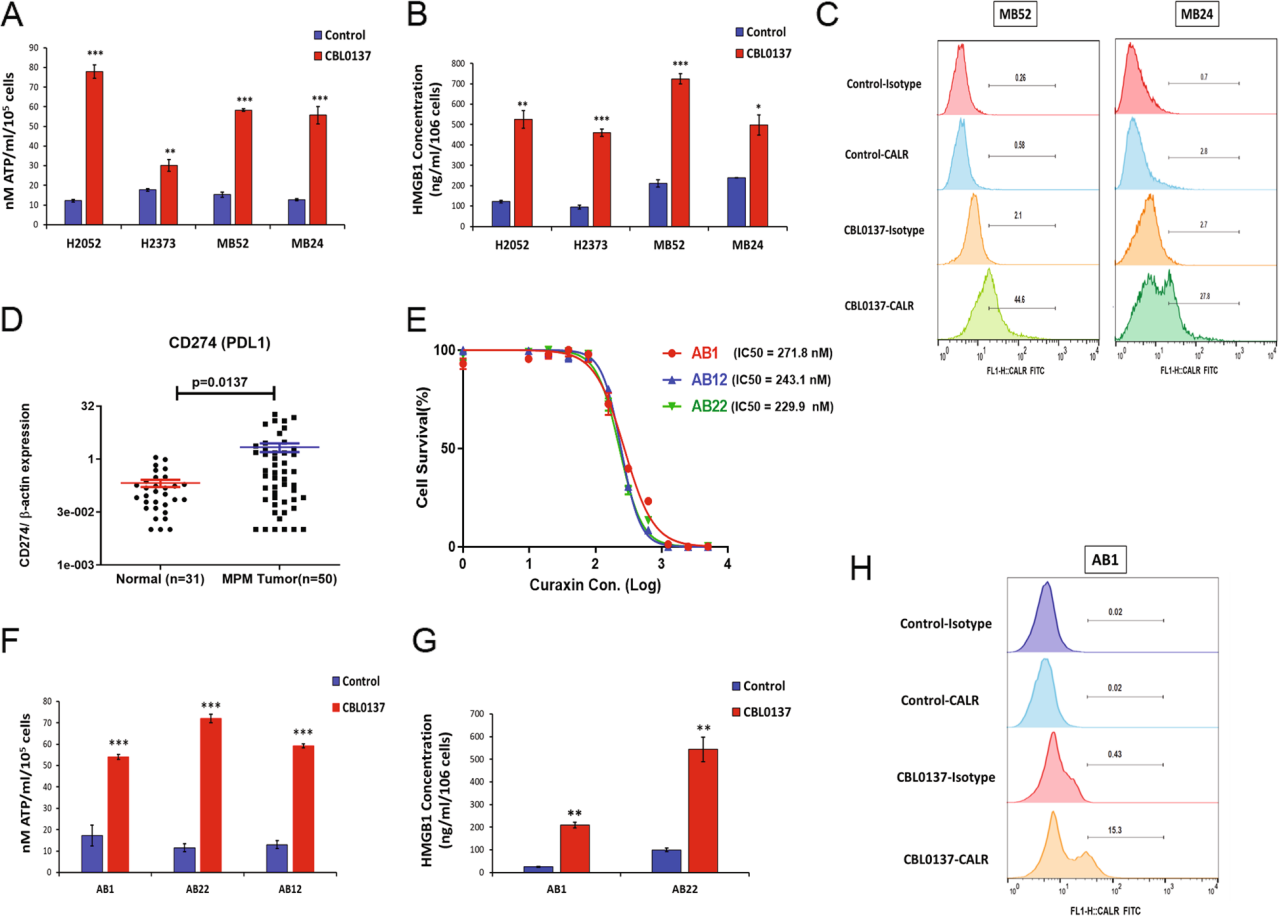
Immunogenic cell death is an additional CBL0137 mechanism of action in mesothelioma cells. **A**-**C** Immunogenic cell death was assessed in DPM cells treated with CBL0137 (300 nM) for 72 h via ATP release (luminescence assay), HMGB1 release (ELISA), and cell surface calreticulin expression (flow cytometry; representative of three independent experiments). **D** CD274 (PD-L1) expression in human tumor and normal tissues was quantified by qRT-PCR. p-values in tissue samples were calculated by the Mann–Whitney test. **E** Graph depicts cell survival of mouse mesothelioma cells (AB1, AB12, and AB22) treated with increasing concentrations of CBL0137 for 72 h. Data are expressed as means ± SD of quadruplicates and are representative of three independent experiments. **F**–**H** Mouse mesothelioma cells were treated with CBL0137 (300 nM) for 72 h and then ATP release, HMGB1 release, and cell surface calreticulin expression were measured (flow cytometry; representative of three independent experiments). Where applicable, data are presented as means ± SEM, *n* = 3. *p < 0.05; **p < 0.01; ***p < 0.001; *p*-value was calculated with the two-tailed Student’s *t* test. PDL1, programmed death-ligand 1; IC50, half maximal inhibitory concentration; CALR, calreticulin

From GSEA, CBL0137 treatment predominantly upregulated toll like receptors (TLR), nod-like receptors (NLR), and RIG-I-like receptors (RLR) signaling which all play a role in anti-tumor immune responses. Subsequently, we hypothesized that CBL0137 may elicit a host anti-tumor response by inducing immunogenic cell death (ICD) [28]. Release of adenosine tri-phosphate (ATP), high mobility group box 1 (HMGB1), and/or cell surface expression of calreticulin (CALR) were measured as surrogates of ICD. CBL0137 treatment significantly increased ATP, HMGB1, and surface expression of CALR in DPM cells compared to control (Fig. 5A-C). Also, in DPM specimens versus unmatched normal pleuras, mean PD-L1 expression was significantly upregulated (Fig. 5D). This PD-L1 pattern was recapitulated in DPM cells (significantly higher) compared to normal mesothelial cells (Supplementary Fig. 7). Furthermore, PD-L1 surface expression was markedly higher in sarcomatoid (H2373 and MB24) compared to epithelioid cells (H2052 and MB52). Overall, these data indicate that CBL0137 induces cell killing effects in DPM that provide rationale for immunotherapy combinations.

To further support this notion, CBL0137 effects were studied in mouse cells (AB1, AB12, and AB22) representing the three histotypes of DPM. CBL0137 treatment at low doses (IC_50_ range 220 to 280 nM) significantly inhibited cell viability (Fig. 5E). This cell killing was accompanied by significantly increased release of ATP and HMGB1 compared to control (Fig. 5F and G), along with upregulated surface expression of CALR (Fig. 5H).

### CBL0137 enhances immunotherapy against DPM in vivo

**Fig. 6:**
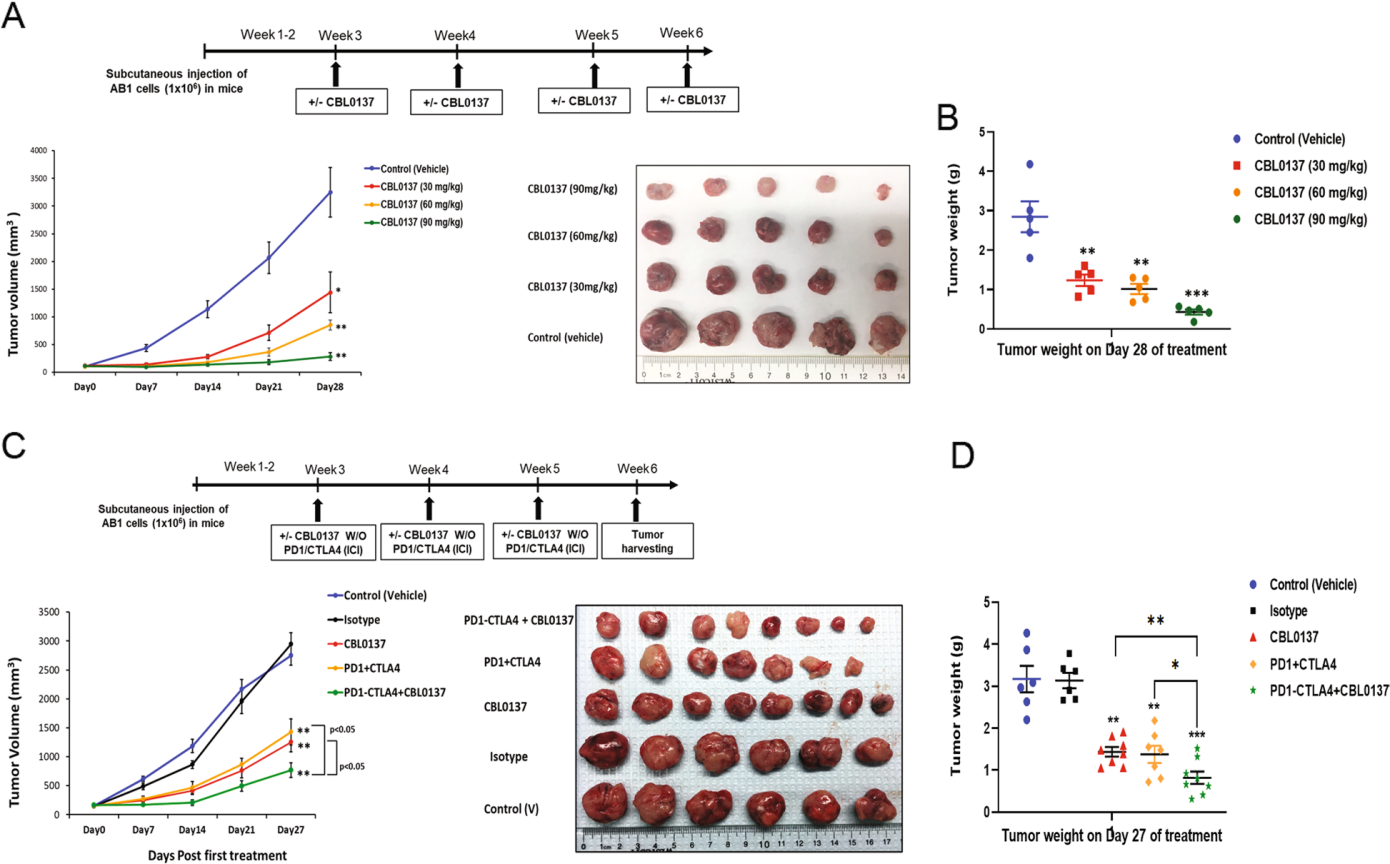
CBL0137 enhances mesothelioma anti-tumor immune response when combined with immunotherapy. **A** The schematic timeline of monitoring tumor growth in BALB/cJ mice bearing tumor allografts during CBL0137 or vehicle control intravenous injection once a week until 4 weeks. AB1 cells were implanted in the flanks to establish subcutaneous tumors. When tumor volume reached an average of 100 mm^3^, mice were randomized and divided in four groups. Mice were treated with different concentrations of CBL0137 or drug vehicle (control) at indicated time points (*n* = 5 in each group). The graph depicts changes in tumor volume over 4 weeks after first injection, and the photo displays the excised tumors. **B** Graph depicts the changes in tumor weight over 4 weeks after first injection. **C** The schematic timeline for combination treatment of CBL0137 with dual ICI in BALB/cJ mice harboring mesothelioma allografts. When tumor volume reached an average of 120 mm^3^, mice were randomized and divided in five groups: drug vehicle (*n* = 6), ICI isotypes (*n *= 6), CBL0137 (*n* = 8), dual ICI (anti-PD1 and anti-CTLA4; *n* = 7), and combination of CBL0137 and dual ICI (*n* = 8). Mice were treated with alternating doses of CBL0137 (20 mg/kg/day via oral gavage twice a week, Days 1 and 2 of week), followed by anti-PD1 (100 µg/mice/day, intraperitoneal) and anti-CTLA4 (50 µg/mice/day, intraperitoneal) on Days 4 and 5 of a week, for a total of three cycles at indicated time points. The graph depicts changes in tumor volume over 4 weeks after first injection, and the photo displays the excised tumors. **D** Graph depicts changes in tumor weight over 4 weeks after first injection. Where applicable, data are presented as means ± SEM. *p < 0.05; **p < 0.01; ***p < 0.001; p-value was calculated with the two-tailed Student’s t or other test. PD-L1, programmed death-ligand 1; PD1, programmed cell death protein; CTLA4, cytotoxic T-lymphocyte-associated protein 4; ICI, immune checkpoint inhibitors

CBL0137 treatment of subcutaneous tumor allografts in a syngeneic murine model significantly suppressed the tumor growth in a dose-dependent manner compared to control group (*p* < 0.05) (Fig. 6A and B). Immunohistochemistry analysis of tumors showed that CBL0137 treatment markedly increased apoptotic cells, PD-L1 expression, and infiltration of CD3^+^ and CD8^+^ T cells compared to control (Supplementary Fig. 8A and B). These results prompted testing CBL0137 combined with ICI. Immunocompetent mice were treated with drug vehicle, CBL0137, isotypes (antibody), anti-PD1 and anti-CTLA-4 antibody, or a combination of CBL0137 with dual ICI at indicated time points. The CBL0137 monotherapy and dual ICI significantly suppressed tumor growth compared to drug vehicle/isotypes (*p* < 0.01), while the combination of CBL0137 plus dual ICI was even more effective (*p* < 0.001) (Fig. 6C). Tumor growth retardation was quantitated by residual weights (Fig. 6D). Taken together, CBL0137 provokes an anti-tumor immune response that can further augment the efficacy of dual ICI therapy, which is currently an approved first-line intervention.

## Discussion

The precise molecular underpinnings of DPM remain obscure, in particular, as to how these tumor genomic features interact to perpetuate malignant behavior [29]. These knowledge gaps, in part, explain the current lack of successful (i.e., FDA-approved) targeted molecular therapies in DPM. There are numerous prior multi-institutional trials which failed to establish clinical adoption of any new targeted drugs against DPM, such as defactinib (COMMAND trial [30]) or nintedanib (LUME-Meso trial [31]), inhibitors of various effectors that mediate receptor tyrosine kinases-Ras-Akt network signals. Yet, there remains an effort to develop other targeted therapeutics. For example, the MiST2 single-arm phase 2 trial provided optimism to further explore the efficacy of cyclin-dependent kinase (CDK)4 and CDK6 inhibitor, abemaciclib, in *CDKN2A*-negative DPM [32].

Our study is similar in concept to these targeted therapies exploiting molecular vulnerabilities of DPM, yet may be more translatable because of the unique mechanism of curaxins. Using murine models, we demonstrate here for the first time that a novel small molecule CBL0137, which inhibits NF-κB while activating p53 tumor suppressor, in low doses, is effective and well tolerated during DPM treatment. This anti-tumor effect is regardless of histotype as the majority of DPM patients harbor wild type *TP53* [8] and active NF-κB pathways [10]. Furthermore, we identify several novel combination regimens, pairing CBL0137 with standard chemotherapy (cisplatin), dual ICI (anti-PD-1 and anti-CTLA-4), and intrapleural short non-coding RNA (miR-215-5p), that are even more efficacious. Each pairing was based on the matching of specific overlapping mechanisms which we verified. Also, a novel insight is that DPM likely depends on FACT complex since the two subunits are significantly overexpressed and the SSRP1 levels are associated with poor prognosis.

Most recently, a phase I trial (NCT01905228) showed that CBL0137 was generally well tolerated in patients (*n* = 83) with advanced solid tumors and, in some, showed anti-tumor activity [33]. Although, the tumor types that showed regression (i.e. best response) were prostate [34] and endometrial [35] cancers, which, counterintuitively, both harbor high rates of *TP53* loss-of-function mutations. Notably, DPM was not evaluated in this study. Hence, our results provide compelling rationale to evaluate CBL0137 for DPM treatment since the mechanism of drug action is well matched with tumor biology. Given the tolerability of CBL0137, p53-targeted therapy may become a clinical reality for DPM patients after all.

In contrast, previous p53 therapeutic strategies have not progressed as part of DPM therapy due to technical and/or clinical factors. For example, adenoviral delivery of *TP53* directly activated anti-tumor effects [36]. Unfortunately, inadequate gene transduction efficiency (low viral infectivity and development of neutralizing antibody) hampered further developments. Additionally, the strategy of using MDM2 inhibitor drugs activating p53 that are biosimilar to nutlin-3a, such as RG7388 (Roche), has been limited by intolerable toxicity profile or by a weak pharmacologic profile [12].

To date, clinical efforts to suppress NF-κB in an anti-DPM approach are not as mature as targeting p53. Little progress has been made since the bortezomib trial failures [14, 15] a decade ago. Later it was recognized that intravenous bortezomib has low penetration and accumulation into solid tumor tissues. This limitation may be overcome, most recently, by intracavitary administration to take advantage of increased local dosing in areas of tumor and decreasing systemic toxicity. In an immunocompetent intraperitoneal murine DPM tumor model, bortezomib suppressed tumor progression and significantly improved survival [37]. Another example of inhibiting NF-κB in DPM relied on using IMD 0354, a selective inhibitor of NF-κB kinase subunit beta (IKKβ) [38]. Immunocompromised mice harboring DPM intrapleural xenografts responded to IMD 0354 as well as, but not superior to, cisplatin therapy, with both agents significantly better compared to control. However, systemic administration of IMD 0354 has not reached human testing.

Perhaps the simultaneous, combinatorial effects on p53 and NF-κB that are core mechanisms in the anti-tumor effect of CBL0137 provide an even more compelling rationale to clinically test this agent, especially against DPM. Additionally, CBL0137 antagonizes multiple other targets important for cancer processes, like *MYC* [17]. Although it is beyond our scope to explore every mechanism, c-Myc, driven by NF-κB in a feed-forward loop, is recognized as a pathogenic factor in DPM [10].

Lastly, a major highlight of our results are the discovery that CBL0137 induces ICD as part of DPM treatment. This mechanism opens the opportunity for novel combinations of chemoimmunotherapy that may achieve durable tumor killing with less toxic side effects. Most intriguing is that the combination of CBL0137 plus dual ICI is effective regardless of tumor histotype and would represent a viable treatment for most DPM patients. This combinatorial strategy should be prioritized as a next step if CBL0137 is confirmed to be effective in a DPM trial. Indeed, this multifunctional capability of CBL0137 impacting multiple diverse molecular targets and its flexibility to effectively combine with diverse drug classes, all may circumvent the resistance that characterizes DPM biology.

## Conclusions

Collectively, our findings support human testing of CBL0137 alone and in combinations, particularly with dual ICI regimens, as a novel therapeutic option for DPM, an otherwise difficult-to-treat tumor.

### Supplementary Information

**Additional file 1: Supplementary Figure 1. **CBL0137 treatment induces apoptosis in DPM Cells. **Supplementary Figure 2. **CBL0137 induces cell cycle arrest in mesothelioma cells. **Supplementary Figure 3. **CBL0137 treatment suppressed the growth of DPM xenografts. **Supplementary Figure 4. **CBL0137 enhances the efficacy of cisplatin and microRNA-215 in DPM.** Supplementary Figure 5. **CBL0137 enhances the efficacy of cisplatin in-vivo. **Supplementary Figure 6. **CBL0137 treatment altered the global gene expression  profiles in DPM cells. **Supplementary Figure 7. **PD-L1 overexpressed in mesothelioma cells. **Supplementary Figure 8. **CBL0137 enhances anti-tumor immune response in DPM. **Supplementary Table 1.  **Histology of MPM cell lines used in this study. **Supplementary Table 2. **Histology of tissue specimens (Hoang’s lab) used for mRNA expression analysis, and TCGA-MESO dataset cohort used for overall survival analysis. **Supplementary Table 4. **List of qRT-PCR TaqMan primer probes (assays) used for gene expression analysis

**Additional file 2: Supplementary Table 3. **Differentially expressed genes after CBL0137 treatment in MB52 and MB24 mesothelioma cell lines by RNA seq

## Data Availability

All datasets used and/or analyzed during the current study are available from the corresponding author on reasonable request.

## Abbreviations

ANOVA: Analysis of variance
ATP: Adenosine triphosphate
CALR: Calreticulin
CBL0137: 1,1′-(9-(2-(Isopropylamino)ethyl)-9H-carbazole-3,6-diyl)bis(ethan-1-one)
CDK: Cyclin-dependent kinase
cDNA: Complementary deoxyribonucleic acid
CTLA-4: Cytotoxic T-lymphocyte antigen-4
DEG: Differentially expressed gene
DMSO: Dimethylsulfoxide
DNA: Deoxyribonucleic acid
FACS: Fluorescence-activated cell sorting
FACT: FAcilitates Chromatin Transcription
FDA: Food and Drug Administration
GSEA: Gene set enrichment analysis
GO: Gene ontology
HMGB1: High mobility group box 1
IC50: Half-maximal inhibitory concentration
ICD: Immunogenic cell death
ICI: Immune checkpoint inhibitor
IKKβ: Inhibitor of NF-κB kinase subunit beta
IRB: Institutional review board
IT: Intrathoracic
IV: Intravenous
KEGG: Kyoto Encyclopedia of Genes and Genomes
MDM2: Murine Double Minute 2
miR: MicroRNA
miRNA: MicroRNA
DPM: Diffuse pleural mesothelioma
mRNA: Messenger ribonucleic acid
MS: Median survival
NF-κB: Nuclear factor kappa-light-chain-enhancer of activated B cells
NIH: National Institutes of Health
NLR: Nod-like receptors
NSG: NOD.Cg-PrkdcscidIl2rgtm1Wjl/SzJ (i.e. NOD-SCID gamma)
OS: Overall survival
PARP: Poly (adenosine diphosphate-ribose) polymerase
PCR: Polymerase chain reaction
PD-1: Programmed death-1
PD-L1: Programmed death ligand-1
qRT-PCR: Quantitative Real-time Polymerase Chain Reaction
RIPA: Radio-immunoprecipitation buffer
RLR: Retinoic acid-inducible gene I-like receptors
RNA: Ribonucleic acid
SD: Standard deviation
SEM: Standard error of the mean
SFH: Surface-fill hydrogel
SSRP1: Structure-specific recognition protein 1
SUPT16H: Suppressor of Ty 16
TCGA: The Cancer Genome Atlas
TLR: Toll like receptors
TNFα: Tumor necrosis factor alpha
