# Supplementary material for: Targeting FAcilitates Chromatin Transcription complex inhibits pleural mesothelioma and enhances immunotherapy
Source: J Exp Clin Cancer Res. 2023 Nov 16;42:304. doi: 10.1186/s13046-023-02889-6 (PMC10652639; doi:10.1186/s13046-023-02889-6)
Supplement: Supplementary file 1 — Additional file 1: Supplementary Figure 1. CBL0137 treatment induces apoptosis in DPM Cells. Supplementary Figure 2. CBL0137 induces cell cycle arrest in mesothelioma cells. Supplementary Figure 3. CBL0137 treatment suppressed the growth of DPM xenografts. Supplementary Figure 4. CBL0137 enhances the efficacy of cisplatin and microRNA-215 in DPM. Supplementary Figure 5. CBL0137 enhances the efficacy of cisplatin in-vivo. Supplementary Figure 6. CBL0137 treatment altered the global gene expression profiles in DPM cells. Supplementary Figure 7. PD-L1 overexpressed in mesothelioma cells. Supplementary Figure 8. CBL0137 enhances anti-tumor immune response in DPM. Supplementary Table 1. Histology of MPM cell lines used in this study. Supplementary Table 2. Histology of tissue specimens (Hoang’s lab) used for mRNA expression analysis, and TCGA-MESO dataset cohort used for overall survival analysis. Supplementary Table 4. List of qRT-PCR TaqMan primer probes (assays) used for gene expression analysis [file 13046_2023_2889_MOESM1_ESM.docx]

**Supplement**

**Figures and Files included**

**Targeting FAcilitates Chromatin Transcription Complex Inhibits Pleural Mesothelioma and Enhances Immunotherapy**

Anand Singh^1^, Nathanael Pruett^1^, Shivani Dixit^1^, Sudheer K. Gara^1^, Haitao Wang^1^, Roma Pahwa^2^, David S. Schrump^1^, and Chuong D. Hoang^1^

^1^Thoracic Surgery Branch

^2^Urologic Oncology Branch

National Cancer Institute, National Institutes of Health, Bethesda, MD, USA;

**Supplementary Figure 1.**


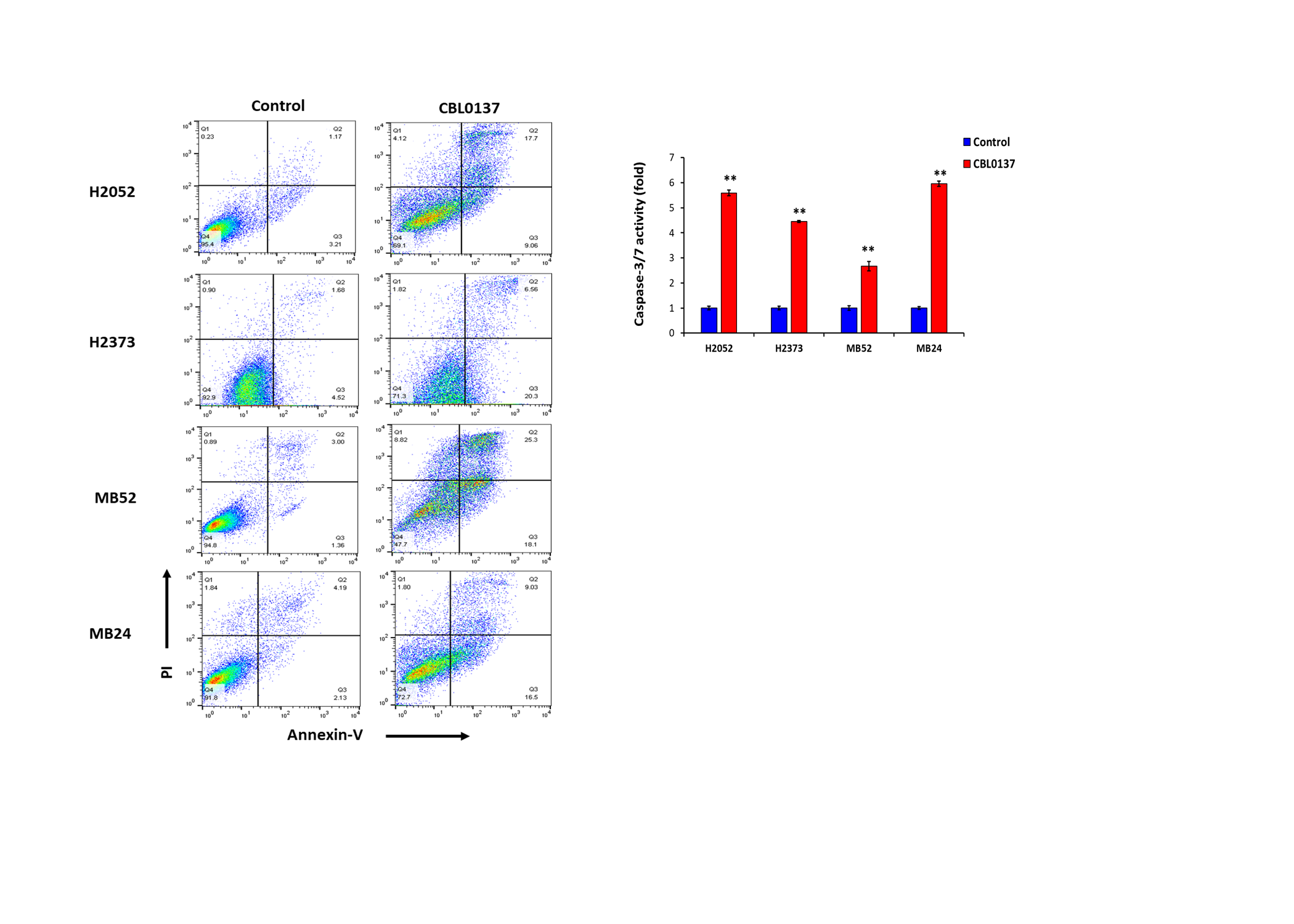
**A B**

**Supplementary Figure 1. CBL0137 treatment induces apoptosis in DPM Cells**

(A) DPM cells treated with CBL0137 (300 nM) compared to control at 72 hours showed significant increase in apoptosis by Annexin-V staining. (B) Caspase3/7 assay exhibited significant increase in caspase-3 activity in DPM cells treated with CBL0137 c compared to control at 72 hours. Data are presented as mean ± SEM. **p < 0.01. p < 0.05 is considered significant and was calculated by the two-tailed Student’s *t* test.

**Supplementary Figure 2.**


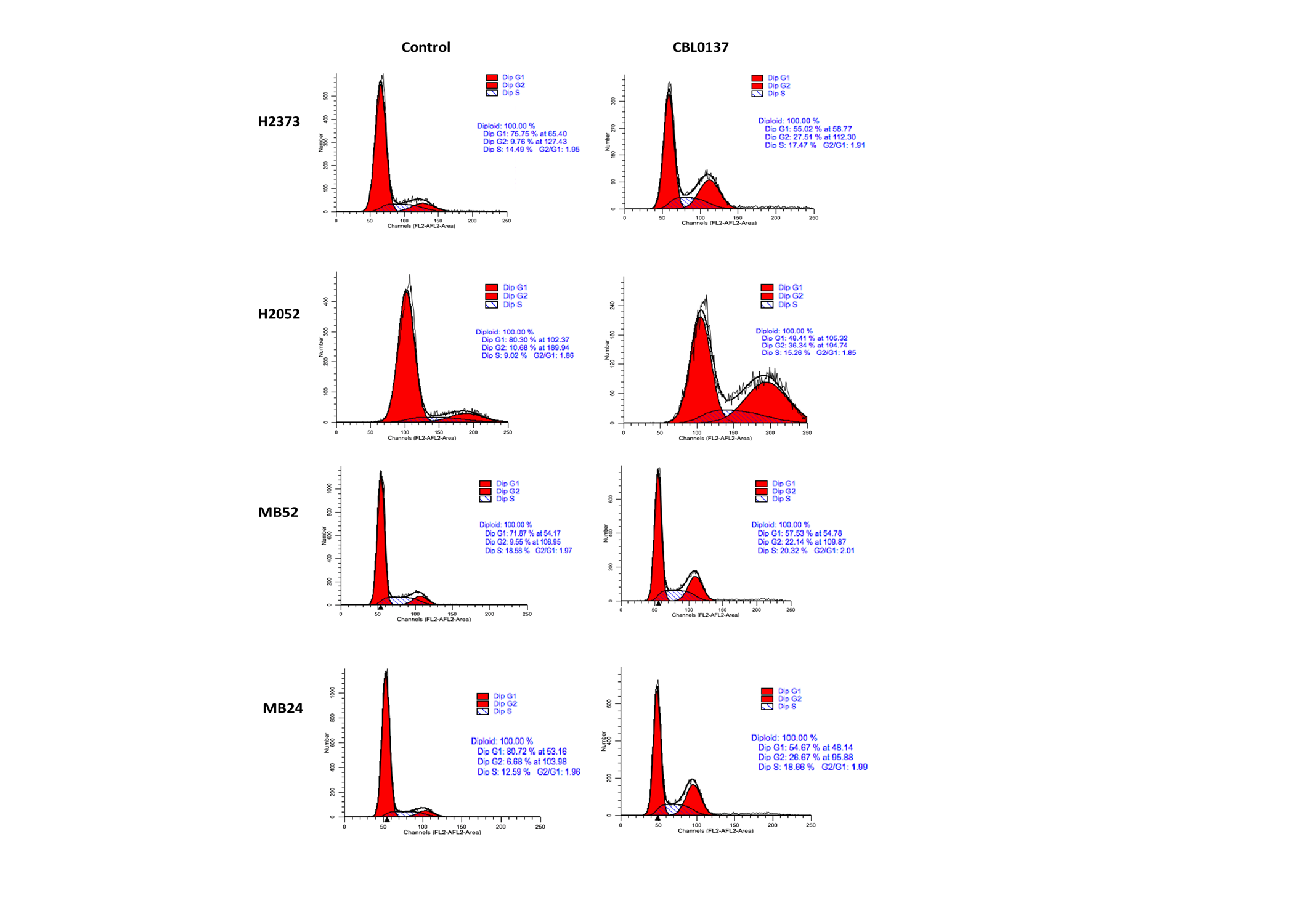


**Supplementary Figure 2. CBL0137 induces cell cycle arrest in mesothelioma cells**

Cell cycle histograms generated from flow cytometry show that CBL0137 (300 nM) treatment significantly arrested the cells in G2-M phase. Data are representative of three independent experiments.


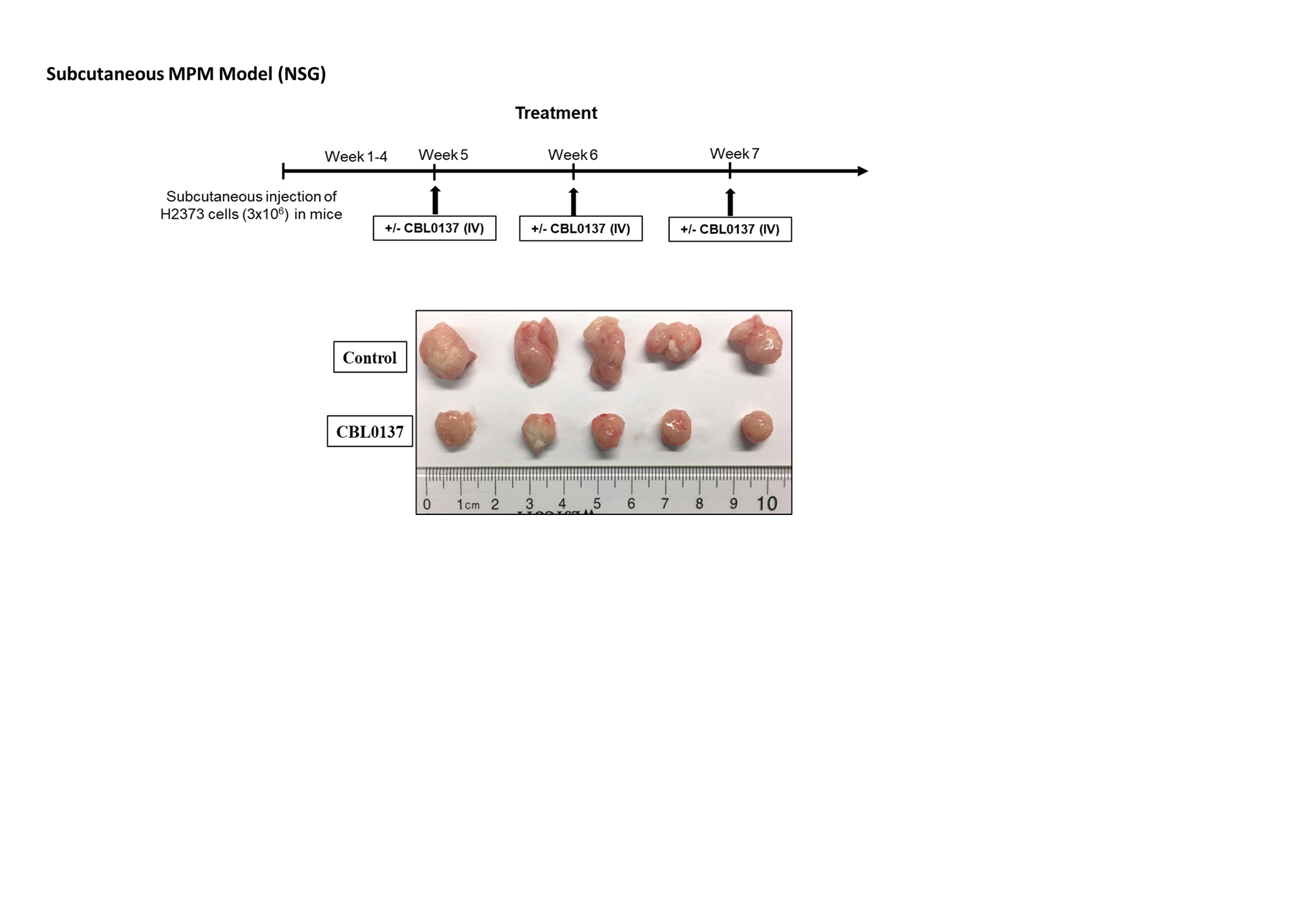
**Supplementary Figure 3.**

**Supplementary Figure 3. CBL0137 treatment suppressed the growth of DPM xenografts**

H2373 cells (3.0 x10^6^) were implanted in the flanks of NSG mice to establish subcutaneous xenografts. Once tumor volume had reached an average of 130 mm^3^, mice were randomized and divided in two groups. Mice groups were injected with CBL0137 or delivery vehicle intravenously (IV) once a week until 3 weeks (n = 5 in each group). Image depicts the excised tumor xenografts after 5 weeks of first drug injection.


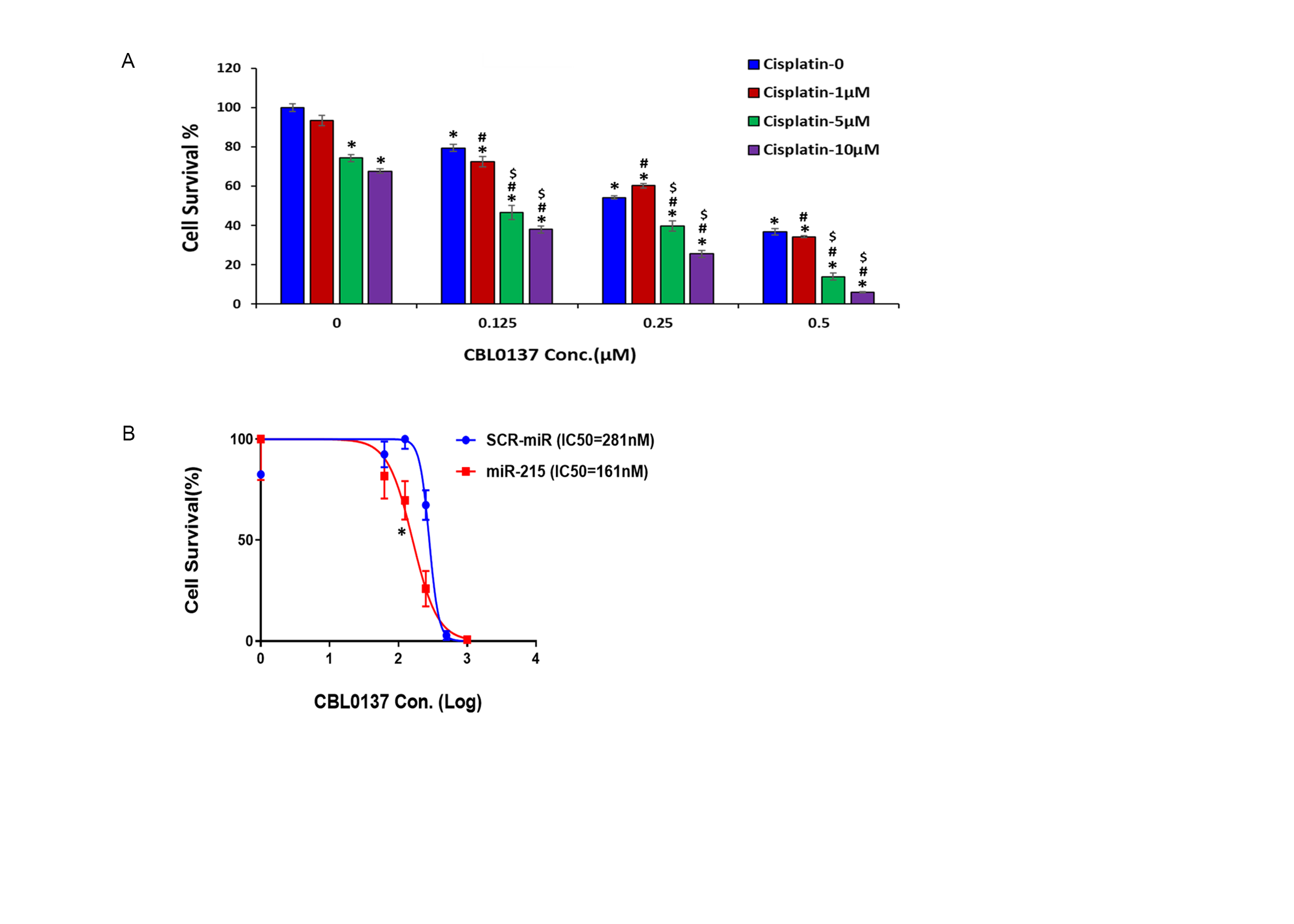
**Supplementary Figure 4.**

**Supplementary Figure 4. CBL0137 enhances the efficacy of cisplatin and microRNA-215 in DPM**

(A) MB52 cells were treated with CBL0137 or cisplatin alone or in combination at different concentrations for 72 hrs. In a cell viability assay, CBL0137 and cisplatin combination exhibited more efficient DPM cell killing at low doses compared to control or monotherapy. (B) miR-215 re-expression potentiates CBL0137 induced cell killing in DPM cells. MB52 cells were transfected with miR-215 or control mimic, and after 24 h, cell survival was measured over increasing concentrations of CBL0137 treatment for an additional 48 h. Data are expressed as means ± SD of quadruplicates and are representative of three independent experiments. data are presented as means ± SEM, n=3. *p < 0.05 (control vs CBL0137/cisplatin/ CBL0137+cisplatin), ^#^p < 0.05 (cisplatin vs CBL0137+ cisplatin**)**, and ^$^p < 0.05 (CBL0137 VS CBL0137+ cisplatin) ; p < 0.05 was considered significant and was calculated with the two-tailed Student’s *t* test.

**Supplementary Figure 5.**


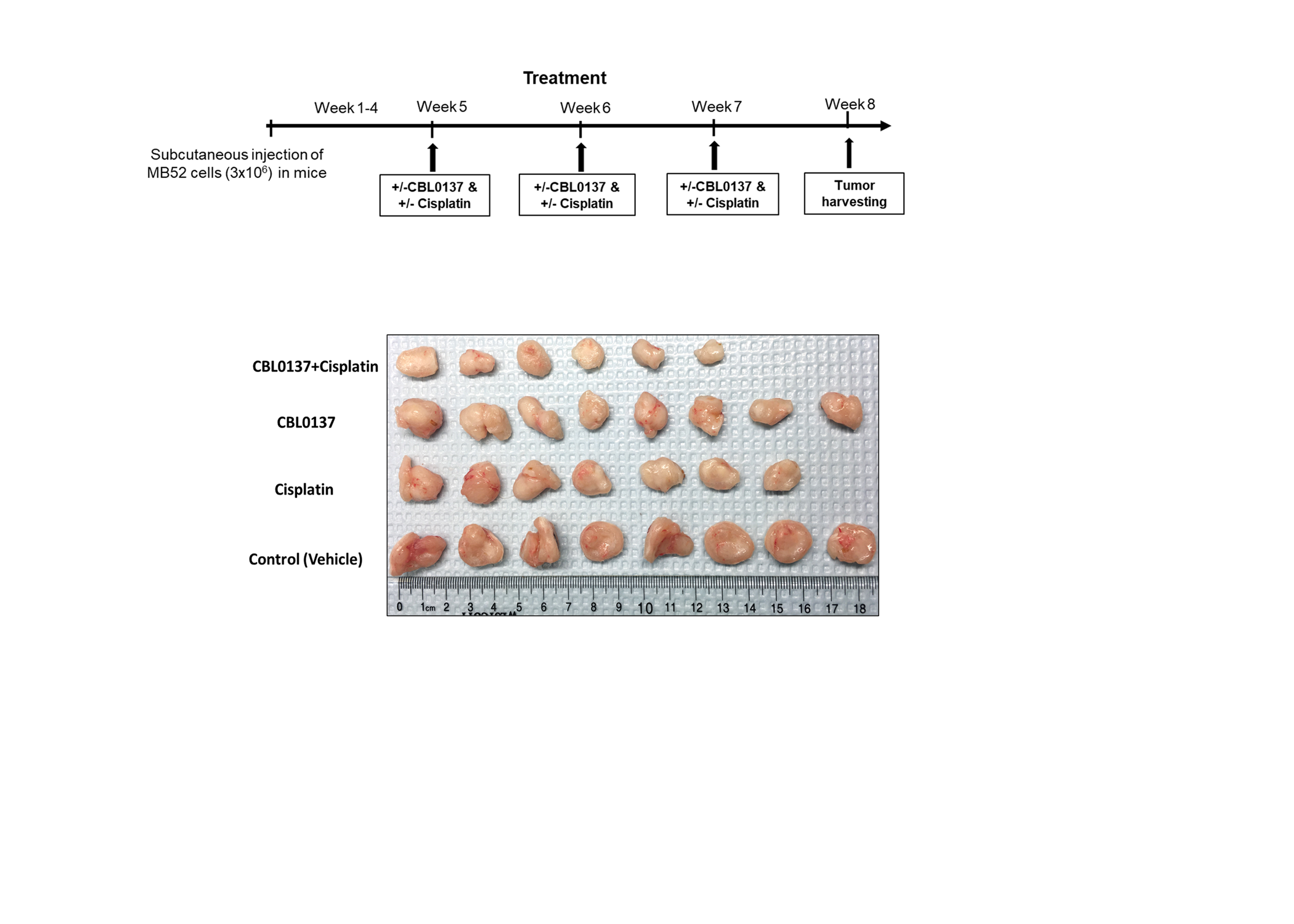


**Supplementary Figure 5. CBL0137 enhances the efficacy of cisplatin *in-vivo***

MB52 cells (3.0 x10^6^) were implanted in the flanks of NSG mice. Once tumor volume reached an average of 120 mm^3^, mice were randomized and divided in four groups. Mice groups were treated with drug vehicle (n=8), CBL0137 (20mg/kg per day via oral gavage, day 1 & 2 of a week, n=8), cisplatin (3mg/kg per day; IP, once a week, n=7), or a combination of CBL0137 and cisplatin (n=6), at indicated time points. Image depicts the excised tumor xenografts after 4 weeks of first drug injection.


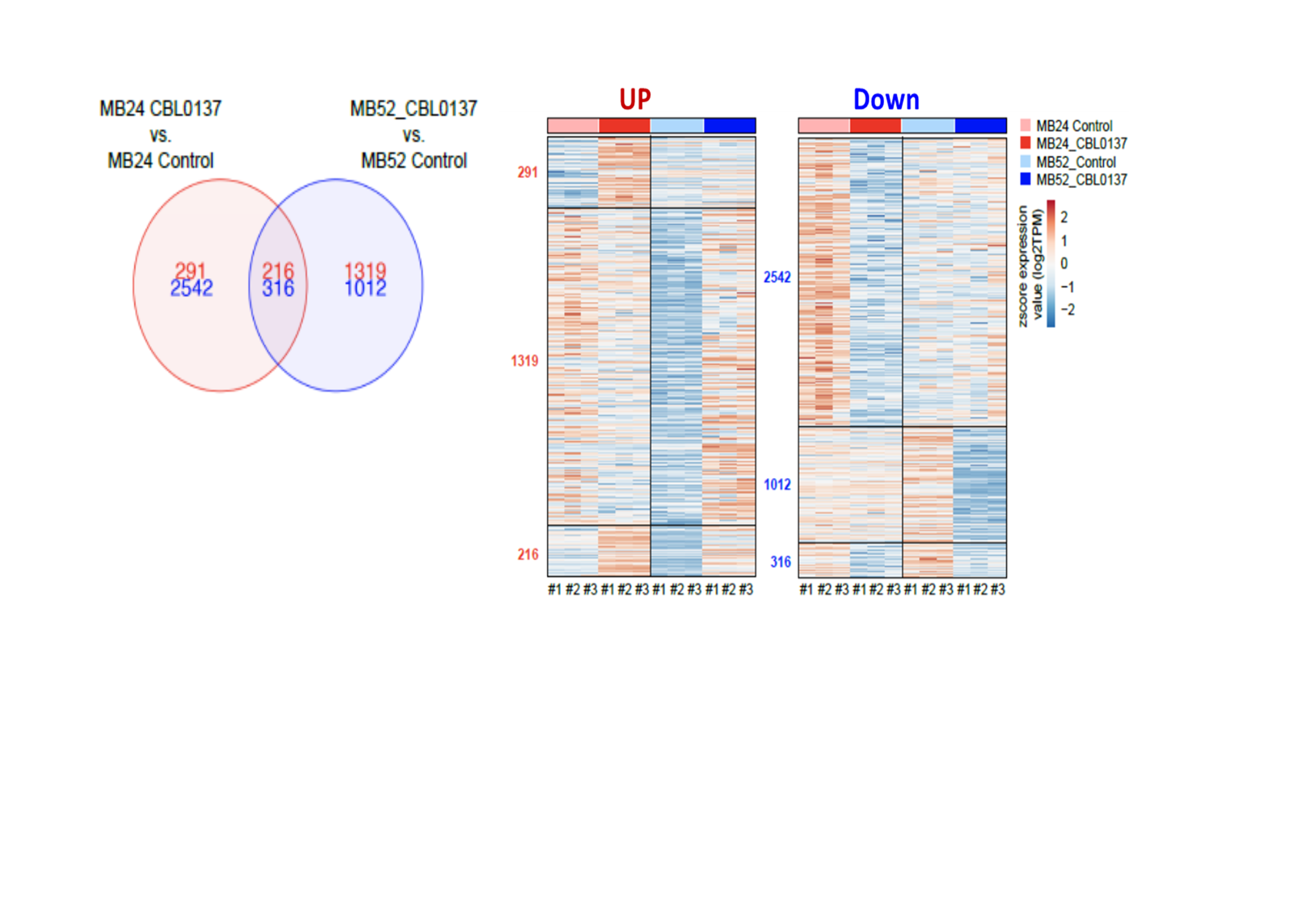
**Supplementary Figure 6.**

**Supplementary Figure 6. CBL0137 treatment altered the global gene expression profiles in DPM cells**

Venn diagram and heatmap showing the number of differentially expressed genes in MB24 and MB52 following treatment with CBL0137 compared to control. The expression of approximately 5,696 genes (1826 up- and 3870 down) was significantly altered (p < 0.05; log_2_ fold change >1) in both DPM cell lines treated with CBL0137 compared to controls. Of the 5,696 differentially expressed genes, only 532 genes were common in both cell lines, 316 genes were downregulated (blue color) while 216 genes were upregulated (Red color).

**Supplementary Figure 7.**


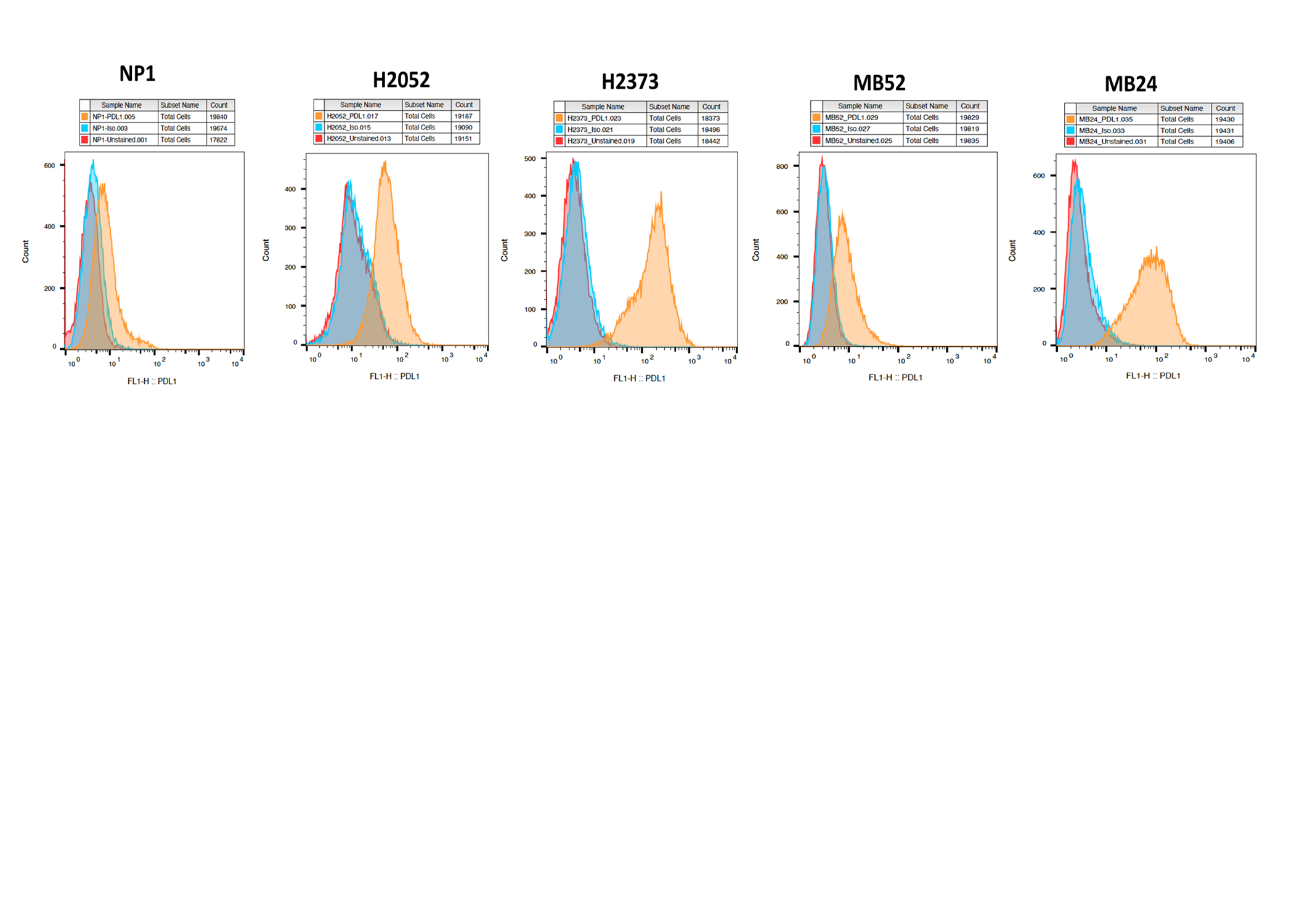

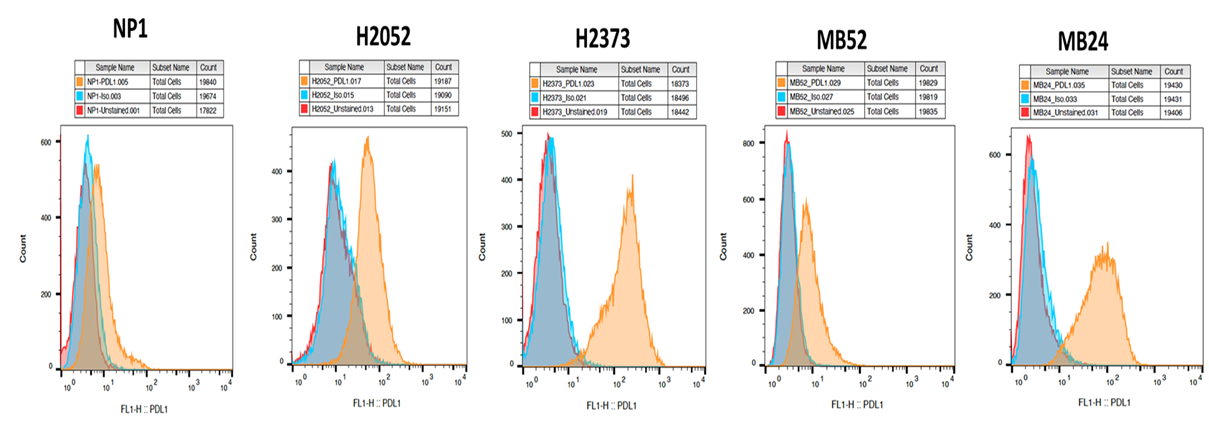


**Supplementary Figure 7. PD-L1 overexpressed in mesothelioma cells**

PD-L1 surface expression was measured in normal mesothelial cells (NP1) and mesothelioma cells ( H2052, H2373, MB52, and MB24) by flowcytometry. Data are representative of three independent experiments.


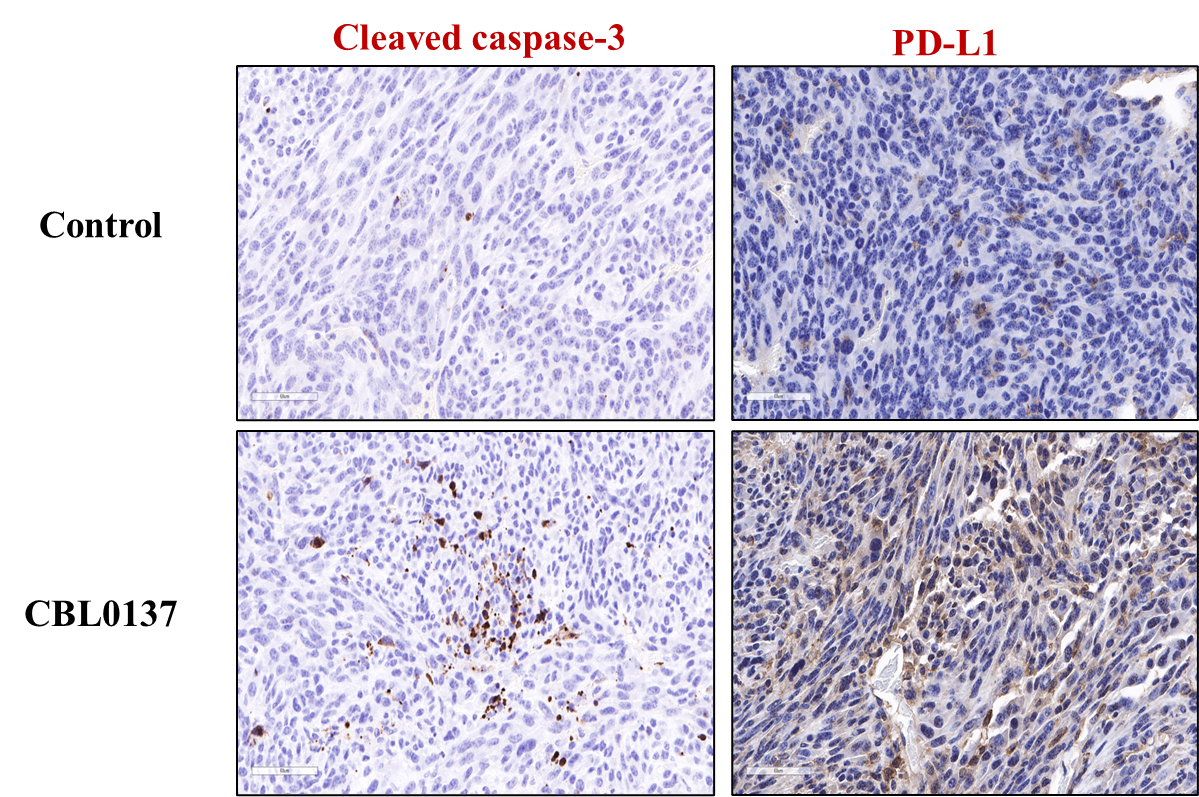
**Supplementary Figure 8.**

**A**

**
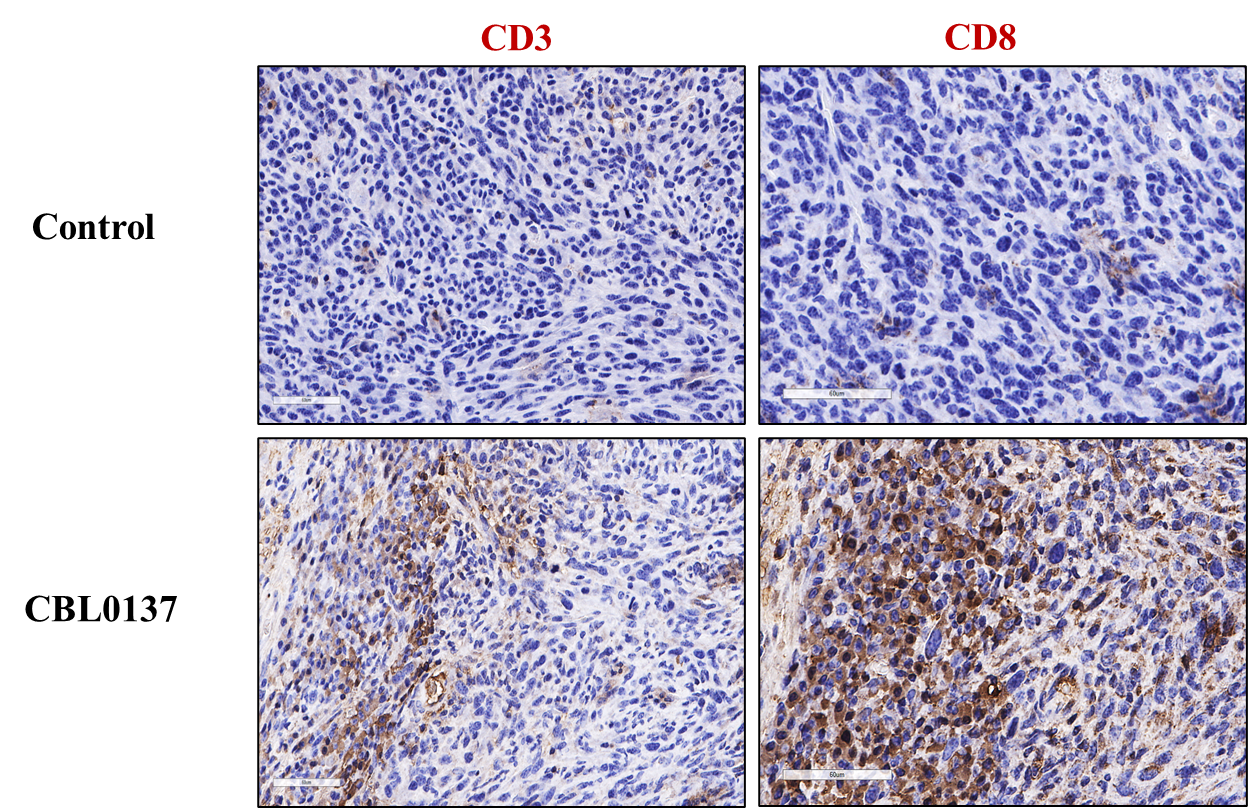
**

**B**

**Supplementary Figure 8. CBL0137 enhances anti-tumor immune response in DPM**

(A) Cleaved caspase-3 levels and PD-L1 expression were assessed by immunohistochemistry in CBL0137 (60 mg/kg) or control treated tumors. Scale bar (white) = 60 μm. (B) Infiltration of CD3+ and CD8+ T cells were measured by immunohistochemistry in CBL0137 (60 mg/kg) or control treated tumors. Scale bar (white) = 60μm.

**Supplementary Table 1**

| **Cell lines** | **Histologic Subtype** |
| --- | --- |
| H2052 | Epithelial |
| H2452 | Epithelial |
| MB52 | Epithelial |
| MB8T | Epithelial |
| H2373 | Sarcomatoid |
| MB24 | Sarcomatoid |
| MB34 | Sarcomatoid |
| MSTO-211H | Biphasic |
| NP1 | Normal mesothelial cells |

Histology of MPM cell lines used in this study.

**Supplementary Table 2**

| **Specimens** | **Number of Specimens (n)** | **Overall Survival data** |
| --- | --- | --- |
| **Hoang Lab (MPM and Pleura)** | **81** | **NA** |
| Epithelioid | 46 | NA |
| Sarcomatoid | 1 | NA |
| Biphasic | 3 | NA |
| Normal Pleura | 31 | NA |
| **TCGA Mesothelioma Cohort** | **87** | **85** |
| Epithelioid | 57 | 55 |
| Sarcomatoid | 2 | 1 |
| Biphasic | 23 | 23 |
| Diffuse Malignant Mesothelioma | 5 | 5 |

Histology of tissue specimens (Hoang’s lab) used for mRNA expression analysis, and TCGA-MESO dataset cohort used for overall survival analysis.

**Supplementary Table 3-file**.**xls**

Differentially expressed genes after CBL0137 treatment in MB52 and MB24 mesothelioma cell lines by RNA seq

**Supplementary Table 4.**

| **TaqMan Assay** | **Catalog Number or Assay ID** | **Manufacture** |
| --- | --- | --- |
| SSRP1 TaqMan expression assay | [**Hs00172629_m1**](https://www.thermofisher.com/taqman-gene-expression/product/Hs00961704_g1?CID=&ICID=&subtype=) | Thermo Fisher Scientific |
| SUPT16H TaqMan expression assay | [**Hs00200446_m1**](https://www.thermofisher.com/taqman-gene-expression/product/Hs01073586_m1?CID=&ICID=&subtype=) | Thermo Fisher Scientific |
| CD274 TaqMan expression assay | [**Hs00204257_m1**](https://www.thermofisher.com/taqman-gene-expression/product/Hs01084828_m1?CID=&ICID=&subtype=) | Thermo Fisher Scientific |
| β-Actin TaqMan expression assay | **Hs99999903_m1** | Thermo Fisher Scientific |

List of qRT-PCR TaqMan primer probes (assays) used for gene expression analysis.
